# Processing Oscillatory Signals by Incoherent Feedforward Loops

**DOI:** 10.1371/journal.pcbi.1005101

**Published:** 2016-09-13

**Authors:** Carolyn Zhang, Ryan Tsoi, Feilun Wu, Lingchong You

**Affiliations:** 1 Department of Biomedical Engineering, Duke University, Durham, North Carolina, United States of America; 2 Center for Genomic and Computational Biology, Duke University, Durham, North Carolina, United States of America; 3 Department of Molecular Genetics and Microbiology, Duke University School of Medicine, Durham, North Carolina, United States of America; Stony Brook University, UNITED STATES

## Abstract

From the timing of amoeba development to the maintenance of stem cell pluripotency, many biological signaling pathways exhibit the ability to differentiate between pulsatile and sustained signals in the regulation of downstream gene expression. While the networks underlying this signal decoding are diverse, many are built around a common motif, the incoherent feedforward loop (IFFL), where an input simultaneously activates an output and an inhibitor of the output. With appropriate parameters, this motif can exhibit temporal adaptation, where the system is desensitized to a sustained input. This property serves as the foundation for distinguishing input signals with varying temporal profiles. Here, we use quantitative modeling to examine another property of IFFLs—the ability to process oscillatory signals. Our results indicate that the system’s ability to translate pulsatile dynamics is limited by two constraints. The kinetics of the IFFL components dictate the input range for which the network is able to decode pulsatile dynamics. In addition, a match between the network parameters and input signal characteristics is required for optimal “counting”. We elucidate one potential mechanism by which information processing occurs in natural networks, and our work has implications in the design of synthetic gene circuits for this purpose.

## Author Summary

From circadian clocks to ultradian rhythms, oscillatory signals are found ubiquitously in nature. These oscillations are crucial in the regulation of cellular processes. While the fundamental design principles underlying the generation of these oscillations are extensively studied, the mechanisms for decoding these signals are underappreciated. With implications in both the basic understanding of how cells process temporal signals and the design of synthetic systems, we use quantitative modeling to probe one mechanism, the counting of pulses. We demonstrate the capability of an Incoherent Feedforward Loop motif for the differentiation between sustained and oscillatory input signals.

## Introduction

From Ca^+2^ signaling to coordination of cell fates, oscillatory signals are essential to regulation of cellular processes [1–4]. The dynamic properties of such signals are crucial for controlling behaviors of single cells and cell populations [5]. As such, the mechanisms underlying the generation of these signals are well-established [2, 6, 7]. For instance, the network constraints governing the circadian clock elucidate design principles dictating the generation of both natural and synthetic pulses [8–10]. Some general requirements for the generation of oscillations include ‘nonlinear’ reaction rates and negative feedback [9]. A systems-level approach to oscillation characterization examines the topologies in natural systems that give rise to pulse generation [9]. This demonstrates the necessity of ‘nonlinear’ kinetic rate laws for the destabilization of the steady state in the generation of oscillations [9]. While this constraint allows the generation of pulses with a diverse set of network motifs, negative feedback (especially negative feedback with a time delay) is found in all these cases. This component is used to reset the network to its initial state [2, 9]. Engineered systems based on such design constraints demonstrate the capability to generate synthetic oscillators mimicking those found in nature [6]. Even in the absence of any apparent regulation, transient oscillations in gene expression can emerge from cell-size control [11].

Despite the ubiquity of oscillations in biology, much less is known about how cells process these signals. In particular, how do cells distinguish between oscillatory and sustained inputs? For a given oscillatory input, how do cells retrieve encoded information from the frequency and amplitude? For signal processing in the frequency domain, computational methods illustrate one potential mechanism, where a critical frequency defines the bandwidth for high fidelity signal propagation for each network [3]. This capacity can be changed with an increased oscillation amplitude or with increased kinetic rates. Regardless of the strategies that give rise to signal encoding, it is important to further understand how cells process oscillatory signals.

**Fig 1:**
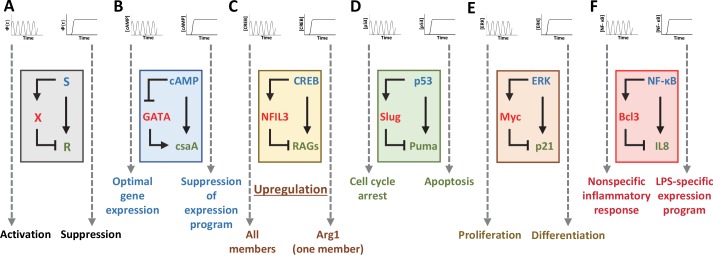
IFFL is a common motif underlying processing of transient or oscillatory signals. (A) An IFFL often underlies signal decoding processes. In an IFFL, an input both activates and represses a single output. (B) GATA in development. Oscillating and sustained cAMP dynamics regulate the developmental program in social amoeba. (C) CREB in regeneration. CREB temporal dynamics control neural regeneration. (D) p53 in cell fate decisions. Pulsatile and sustained p53 stimulation results in either cell cycle arrest or apoptosis respectively. (E) ERK in cell fate decisions. Differentiating between pulsatile and sustained ERK stimulation is required for the regulation of stem cell differentiation and proliferation. (F) NF-κB in immune responses. Differentiating between pulsatile and sustained NF-κB stimulation is required for the induction of non-specific inflammatory responses and LPS-specific expression programs.

Many natural biological networks exhibit the ability to distinguish oscillatory and sustained signals. While several studies describe the contrasting downstream phenotypes, the architectures that give rise to such outcomes remain unclear. One common motif shared by such networks is the Incoherent Feed-Forward Loop (IFFL), in which an input both activates and represses a single output (Fig 1A) [4, 12, 13]. For example, oscillations in the transcription factor Ascl1 play a critical role in driving the proliferation of multipotent neural progenitor cells (NPCs) [14, 15]. In contrast, the sustained expression of Ascl1 promotes neuronal fate differentiation in NPCs [15, 16]. In social amoeba *Dictyostelium discoideum*, 3’ 5’-cyclic adenosine monophosphate (cAMP) oscillations result in the optimal gene expression for development while continuous stimulation inhibits transcription (Fig 1B) [17–19]. cAMP directly induces the expression of contact site A gene (csaA) while repressing the transcriptional activity of GtaC on csaA [17]. Additionally, the number of cAMP pulses regulates the coordination of development.

An IFFL also underlies the regulation of neuronal regeneration by cAMP response element binding protein (CREB). CREB activates regeneration-associated genes (RAGs) and nuclear factor interleukin-3-regulated protein (NFIL3), which negatively regulates RAGs (Fig 1C) [20]. In Drosophila, the constitutive activation of CREB upregulates a single RAG, Arg1, while CREB oscillations induced by neuronal injury increase the transcription of all members [20, 21].

The IFFL motif is also found downstream of p53 in the regulation of cell fate decisions. The tumor suppressor p53 oscillates in response to γ-radiation, and the number of oscillations increase the level of damage. In contrast, UV radiation induction results in a sustained pulse of p53 [22]. While p53 oscillations lead to cell cycle arrest, sustained p53 activation induces apoptosis [23, 24]. Here, p53 activates P53 upregulated modulator of apoptosis (PUMA), which plays a role in the induction of apoptosis, and Slug, a transcription factor which represses the activity of PUMA (Fig 1D) [25].

In mammalian cells, pulsatile dynamics of extracellular signal-regulated kinases (ERK) in response to epidermal growth factor (EGF) result in proliferation [26–28]. However, nerve growth factor (NGF) induces sustained ERK, driving differentiation [16]. Here, the transcription factor Myc and the cyclin-dependent kinase inhibitor p21 are both activated by ERK [29, 30]. Myc, an inducer of proliferation, represses the activity of p21, an inhibitor of cell cycle progression (Fig 1E) [31–35].

Tumor necrosis factor-α (TNFα) can stimulate oscillations in the transcription factor NF-κB, which in turn triggers nonspecific inflammatory response genes in immune cells [36]. In addition, persistence of the NF-κB oscillations dictates the resulting transcriptional profile [37, 38]. In contrast, bacterial liposaccharides (LPS) promote sustained NF-κB activation, which induces a LPS-specific expression program [39]. We identify a potential IFFL where NF-κB activates the expression of both IL8, a cytokine, and BCL3, a competitor for NF-κB target promoters including IL8 (Fig 1F) [40].

The common occurrence of IFFLs in these networks suggests a role in processing transient or oscillatory signals. This motif is ideal for decoding oscillatory signals due to its ability to react to fold-changes rather than the absolute concentration of an input [12, 13, 41, 42]. In addition, IFFLs are one of the major core topologies that can drive temporal adaptation, the generation of a pulse in response to a sustained input [43]. In essence, this network is able to desensitize a system to a sustained stimulus while maintaining the ability to respond to periodic stimulation. This property is the fundamental principle behind the ability to differentiate pulsatile and sustained signals.

Here, we examine a mechanism by which IFFLs process oscillations and the conditions that facilitate counting of pulses. This network exists in two states—one in which the system resets when the counting mechanism fails due to a conflict between the signal requirements and the actual input, and one where pulses result in a stepwise increase of the output. To differentiate between sustained and oscillatory signals, the network must display both states. The system must exhibit a stepwise increase of the output in response to oscillatory signals while having no response to sustained signals. Analogous to the use of radios to transform information from the frequency and amplitude domains of electromagnetic waves, the IFFL acts as a decoder, recognizing pulsatile signals.

## Results

**Fig 2:**
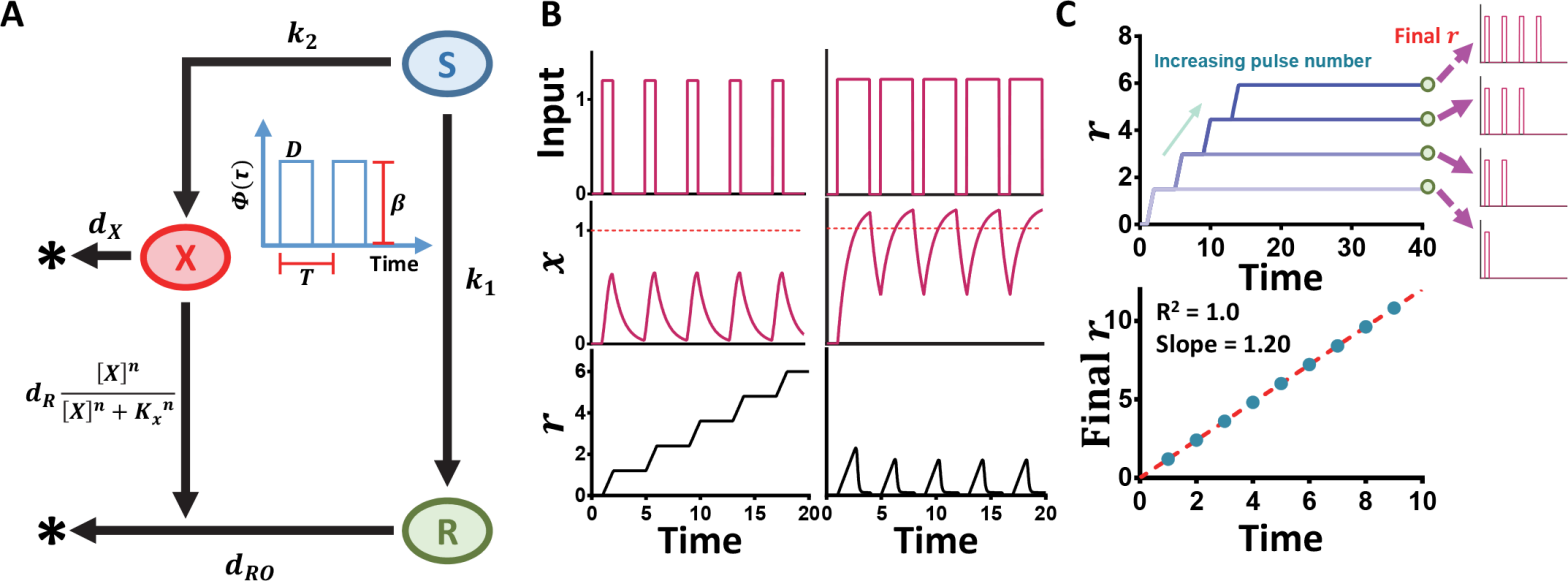
Processing of oscillating signals by an IFFL. (A) Signal processing by an IFFL. In this model, a pulsing input (*Φ*(*τ*)) simultaneously stimulates the production of an intermediate (*X*) and a reporter (*R*). *X* induces the degradation of *R* through a threshold response. The fundamental constraints for counting shown are based on the full model (Methods) while Eqs 1 and 2 are the non-dimensionalized form of the model. Here, the duration (*D*) represents the length of the pulse while the cycle period (*T*) represents the time between the start points of two adjacent pulses. The specific parameters of the IFFL limits the duration range for which counting can occur; only sufficiently short pulses are counted while pulses of a longer duration will shut down the system. (B) Time courses demonstrate counting mechanism. Using *β* = 1.2, *γ*_*R*_ = 10, *γ*_*O*_ = 0, and *T* = 4 (Left: *D* = 1 Right: *D* = 3). The top row contains time courses of the input pulses for two different pulse durations, either a pulsing input or a simulated sustained input. The second row shows time courses for *X*. The bottom row shows time courses for *R*. With identical parameters, an IFFL motif can generate two distinct outputs depending on the length of the duration of the input pulses. When *x* is below the threshold of induction, the circuit maintains the ability to produce a stepwise increase of *r*. However, when *x* overcomes the threshold, the circuit loses this ability. For a single parameter set, both outputs are desired to differentiate between oscillating and sustained signals. This ability is maintained in the case of an input pulse with the form of a sine function (S1 Fig). (C) Calibration curve for ideal counting. Using *β* = 1.2, *γ*_*R*_ = 10, *γ*_*O*_ = 0, and *T* = 4 (*D* = 1) The sample calibration curve is for a pulse duration within the optimal duration range, therefore it is able to demonstrate ideal counting. With an increasing number of input pulses from 1–4 in the top panel, *R* exhibits a stepwise increase. The linearity is demonstrated in the bottom panel by R^2^ ≥ 0.99.

We model an IFFL consisting of three components (Fig 2A): an input node (***S***), an intermediate node (***X***), and an output node (***R***). ***S*** activates the production of both ***X*** and ***R***, while ***X*** induces the degradation of ***R*** through Hill kinetics. The dynamics of the motif can be described with two dimensionless ordinary differential equations (ODE):

$$\frac{dr}{d\tau }=\beta \Phi (\tau )-{(\gamma }_{R}\frac{x^{n}}{x^{n}+1}+\gamma_{o})r,$$ (1)

$$\frac{dx}{d\tau }=\beta \Phi (\tau )-x,$$ (2)

where ***Φ***(***τ***) represents the input signal, which can be sustained, oscillatory, or transient. Unless noted otherwise, we define ***Φ***(***τ***) as a periodic square wave function with ***k*** pulses: ***Φ***(***τ***) = **1** when ***iT*** + ***τ***_***o***_ ≤ ***τ*** < ***iT*** + ***D*** + ***τ***_***o***_ and ***Φ***(***τ***) = **0** otherwise. Here, ***i*** is the index of a pulse (**0** ≤ ***i*** < ***k***), ***τ***_***o***_ is the start time of the first pulse, ***T*** is the cycle period, and ***D*** (< ***T***) is the pulse duration. For all simulations we assume that ***τ***_***o***_ = **1**. ***x*** denotes the concentration of ***X***, ***r*** denotes that of ***R***, and ***τ*** denotes time. ***γ***_***o***_ represents the basal degradation rate constant of ***R***, ***γ***_***R***_ is the maximal induced degradation rate of ***R*** by ***X***, and ***β*** is the maximal synthesis rate of ***X*** and ***R***. We require that the induced degradation of ***R*** have an ultrasensitive dependence on ***X***, as indicated by a high Hill coefficient (***n*** = **110**). Such ultrasensitive dependence can be achieved with oligomerization, tandem binding sites, increased cascade lengths, covalent modification cycles, or titration by inhibitors [44–48]. For titration, competitive binding of the inhibitor sets an activation threshold to be overcome, thereby increasing the Hill coefficient.

We consider ***Φ***(***τ***) with varying durations and cycle periods: a longer duration corresponds to a more sustained signal. We first consider the case where the degradation of ***R*** is solely induced by ***X*** (***γ***_***o***_ = **0**). Fig 2B illustrates typical time courses of ***X*** and ***R*** in response to two different oscillatory signals. In either case, ***X*** peaks in response to each individual pulse and returns to a basal level before the subsequent pulse. When the duration is sufficiently small (Fig 2B: left column), ***r*** exhibits a linear stepwise increase with an increasing number of pulses (for a constant **β** and pulse duration). For a long duration (Fig 2B: right column), however, ***R*** exhibits transient pulses similar to that of ***X***. That is, only an input with a sufficiently short duration generates a more sustained output. The critical determinant of these divergent outcomes is the dynamics of ***X***, which triggers the effective degradation of ***R*** when it reaches its half-activation threshold (***x*** = **1**). For a sufficiently short duration, ***x*** never reaches **1**, and thus is unable to trigger the degradation of ***R***. As a result, ***r*** increases with each additional input pulse. In contrast, for a long duration, ***x*** exceeds **1** for each pulse, leading to the periodic resetting of ***R***.

For an input signal with short pulses, ***R*** can count the number of pulses: the level of ***R*** at a fixed time point (***r***(***τ***_***E***_)) is approximately proportional to the number of pulses for a fixed pulse duration and amplitude (Fig 2C). A least-squares linear regression on ***r***(***τ***_***E***_) versus the number of pulses provides a quantitative measure for the quality of counting. The slope of this line measures the strength of the response. For instance, the slope is near zero for the signal with long pulses (S2 Fig), indicating no response (Fig 2B). The quality of the fit, as measured by **R**^**2**^, quantifies the performance of counting. In all our simulations, we consider **R**^**2**^ **= 0.99** as the cut-off for high-quality counting. With **0 < R**^**2**^ **< 0.99**, low-quality counting emerges as ***r*** saturates (reaches a steady state) over time. This characteristic impairs the ability to predict ***r***(***τ***_***E***_) with an increasing number of pulses. In addition, the absolute failure of counting occurs when **R**^**2**^ **= 0**.

**Fig 3:**
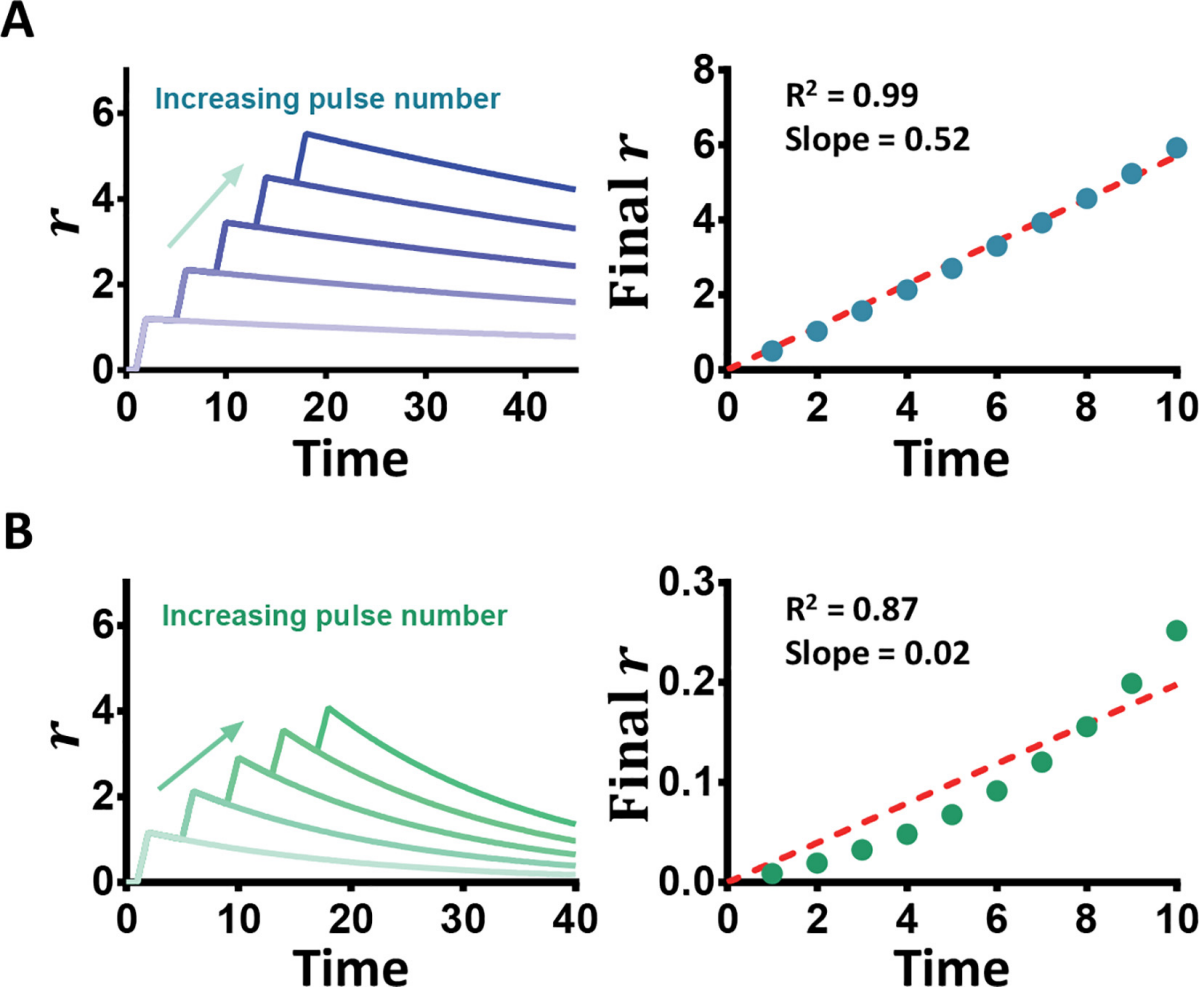
γ_o_ mediates counting quality. Using *β* = 1.2, *γ*_*R*_ = 10, *T* = 4, and *D* = 1, time courses are shown to demonstrate cases of high quality counting and poor counting through the manipulation of *γ*_*o*_. (A) High Quality counting (R^2^ ≥ 0.99) occurs in the case of a small *γ*_*o*_ (*γ*_*o*_ = 0.01), as characterized by the linearity of the calibration curve composed of the final *r* of between 1 and 10 pulses. (B) In the case of a large *γ*_*o*_ (*γ*_*o*_ = 0.05), the nonlinearity of the calibration curve and small slope demonstrate low quality counting (0 < R^2^ < 0.99).

In the base model (Fig 2), we assume that ***R*** is degraded solely by ***X***. In general, however, a basal level degradation of ***R*** can deteriorate the counting quality (Fig 3A). A highly unstable reporter (***γ***_***o***_ = **0.05**; Fig 3B) decreases the counting quality such that **R**^**2**^ **< 0.99**. The decreased slope also indicates the weakened response to the oscillating input. This loss of counting is attributed to the decreased timespan for which ***r*** can be maintained in the absence of the input. Therefore, to reduce the impact of endogenous degradation of the reporter on the counting quality, ***γ***_***o***_ can be minimized through the use of a highly stable reporter.

**Fig 4:**
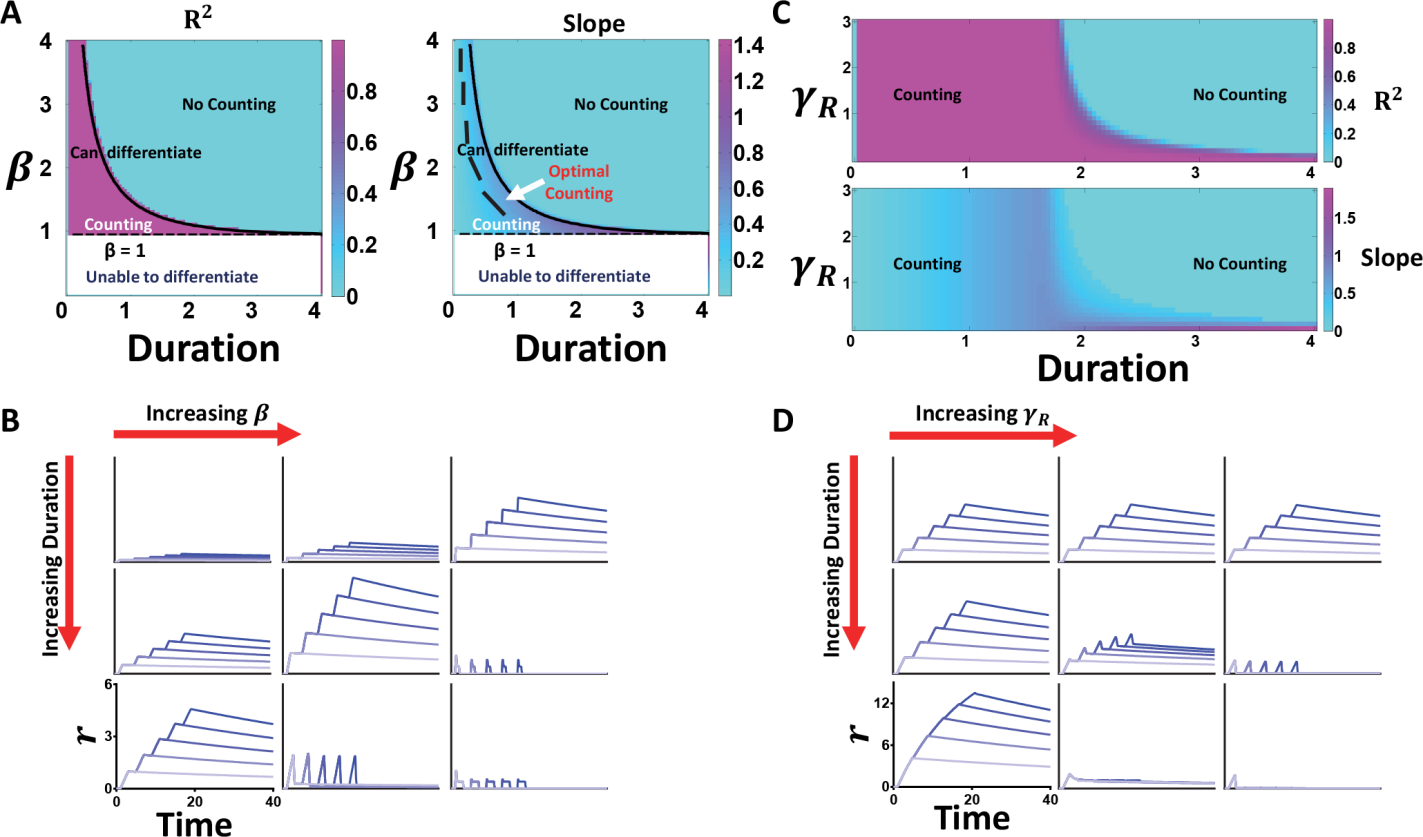
Dynamic constraints underlying reliable counting. Using *γ*_*O*_ = 0.01, *γ*_*R*_ = 10, *T* = 4, *β* = 0–4 (Panels A and B). (A) Effects of varying *β* and duration on R^2^ and slope. Simulation results for the R^2^ and slope of the calibration curve as both *β* and the pulse duration changes from 0–4 with a constant period of 4. From the graph of the R^2^ values, below the analytical border (solid line), counting is maintained (predominantly R^2^ ≥ 0.99), but is lost above this border (R^2^ = 0). From the graph of the slope, optimal counting (region with the highest slope) lies between the analytical border and the dashed line. In both plots, the region under the dotted line (at *β* = 1 in white) is where the IFFL cannot differentiate between pulsing and sustained inputs. (B) Effects of varying *β* and duration on time courses. Time courses with varying number of pulses demonstrate the range of counting for a changing *β*. As *β* increases, the ability to differentiate between different numbers of pulses increases while the range of counting decreases. Using *γ*_*O*_ = 0.01, *β* = 1.2, *T* = 4, *γ*_*R*_ = 0–3 (Panels C and D). (C) Effects of varying *γ*_*R*_ and duration on R^2^ and slope of calibration curves. Simulation results for the R^2^ value and the slope of the calibration curve as *γ*_*R*_ changes from 0–3 and the pulse duration changes from 0–4 with a constant period of 4. As *γ*_*R*_ decreases, the network loses the capability to distinguish between pulsing and sustained inputs. (D) Effects of varying *γ*_*R*_ and duration on time courses. Time courses with different number of pulses demonstrate the range in the counting capability for a changing *γ*_*R*_. As *γ*_*R*_ increases, the range of the counting capability decreases.

Relaxing this assumption, we reach the same conclusions for reliable counting when ***γ***_***o***_ is sufficiently small: our simulations demonstrate that the counting capability can be maintained in the general condition **(*γ***_***o***_ **> 0)**, like in the base case (Fig 4). IFFL kinetics constrains the ability to process oscillatory signals; therefore, we predict that both the production rate (***β***) and the induced degradation rate of the output (***γ***_***R***_) are core parameters. In Fig 4A, we evaluate the range of the counting capability with respect to two parameters, ***β*** and ***D*** (sample time courses shown in Fig 4B). Even in the presence of basal degradation (***γ***_***o***_ = **0.01**), counting can be maintained for appropriate combinations of ***β*** and ***D***. We observe that as ***β*** increases, the range of durations for which counting occurs decreases while increasing the slope for different numbers of pulses (for **R**^**2**^ **≥ 0.99**). On the other hand, increasing ***D*** increases the slope in cases when **R**^**2**^ **≥ 0.99** due to the increased time for ***R*** production.

To differentiate between pulsing and sustained inputs, ***X*** must overcome the activation threshold in the case of a sustained input. Therefore, the network cannot discern signals when ***β*** ≤ **1** because the threshold can never be reached. In this case, the network is unable to display the two necessary states to discern signals, illustrated by **R**^**2**^ **≥ 0.99** at all pulse durations (Fig 4A). However, when ***β*** > **1**, the system has the potential to discriminate inputs (illustrated by a range of durations where **R**^**2**^ **= 0** and a range where **R**^**2**^ **≥ 0.99**) and the pulse duration restricts the counting capability. To count, the duration of input’s OFF state in each cycle (***T*** − ***D***) needs to be long enough to reset the basal level of the intermediate such that the subsequent pulse will not push ***x*** to **1**. Generally, shorter pulse durations are more likely to exhibit this capability due to the longer duration of the OFF state (Fig 4B). The boundary between counting and no counting is determined by whether each pulse can drive ***X*** to the threshold, leading to complete degradation of ***R***. The level of ***X*** at the end of the first pulse is (**1** − ***e***^−***D***^). Therefore, the boundary between the two regions is set by $\beta =\frac{1}{1-e^{-D}}$, which approximates the border observed in numerical simulations (Fig 4A).

For a small ***γ***_***o***_, another critical parameter for counting is the rate constant for induced degradation, ***γ***_***R***_. Fig 4C and 4D depicts the case when ***β*** > **1** with a varying ***γ***_***R***_ and pulse duration for a constant cycle period. With a constant ***β***, we expect the counting range to remain constant. Instead, numerical simulations demonstrate a monotonically decreasing trend of the counting range as ***γ***_***R***_ increases. Above a point, increasing ***γ***_***R***_ has no impact on counting because a higher ***γ***_***R***_ provides no additional benefit on the ability to degrade ***R*** in the case of a sustained input. Below this point, however, the range of pulse durations that can count increases, but the ability to differentiate pulsing and more sustained inputs diminishes due to a higher range of low-quality counting (**0 < R**^**2**^ **< 0.99**). For a sufficiently low ***γ***_***R***_, the system cannot discriminate between the two types of inputs (Fig 4C). We attribute this characteristic to the decreased impact of the intermediate on the degradation of the reporter, which is required for maintaining temporal adaptation.

The kinetics of the system prevent the complete degradation of ***x*** to **0** after each pulse, leading to the progressive accumulation of ***X*** with each additional pulse. Starting from a basal level, ***X*** reaches the maximum concentration (peak) at the end of the pulse, and returns to a new basal level before the next pulse:

$$x_{basal,\;i}=x_{peak,\;i-1}e^{-(T-D)},$$ (3)

$$x_{peak,\;i}=\beta -(\beta -x_{basal,\;i})e^{-D},$$ (4)

where ***x***_***peak*, *i***_ is the peak concentration of ***X*** after the ***i***^***th***^ pulse, ***x***_***basal*, *i***_ is the basal concentration of ***X*** before the ***i***^***th***^ pulse, and ***T*** = cycle period. To start, ***x***_***peak*, 0**_ = **0** while both ***x***_***basal*, *i***_ and ***x***_***peak*, *i***_ increase alongside ***i***. Counting fails at pulse ***nc***, when ***x***_***peak*, *nc***_ > **1**. We term ***nc*** the counting capacity of an IFFL for a given input signal, which sets the maximum number of pulses the network can count. As ***nc*** approaches ∞, the network can maintain counting when the negligible accumulation of ***X*** allows for indefinite counting. This exists when the pulse duration satisfies $\frac{1-e^{-D}}{1-e^{-T}}<\frac{1}{\beta }$. In addition, as ***T*** approaches ∞, the analytical border converges to **1** = ***β***(**1** − ***e***^−***D***^) such that counting is maintained when **1** > ***β***(**1** − ***e***^−***D***^).

**Fig 5:**
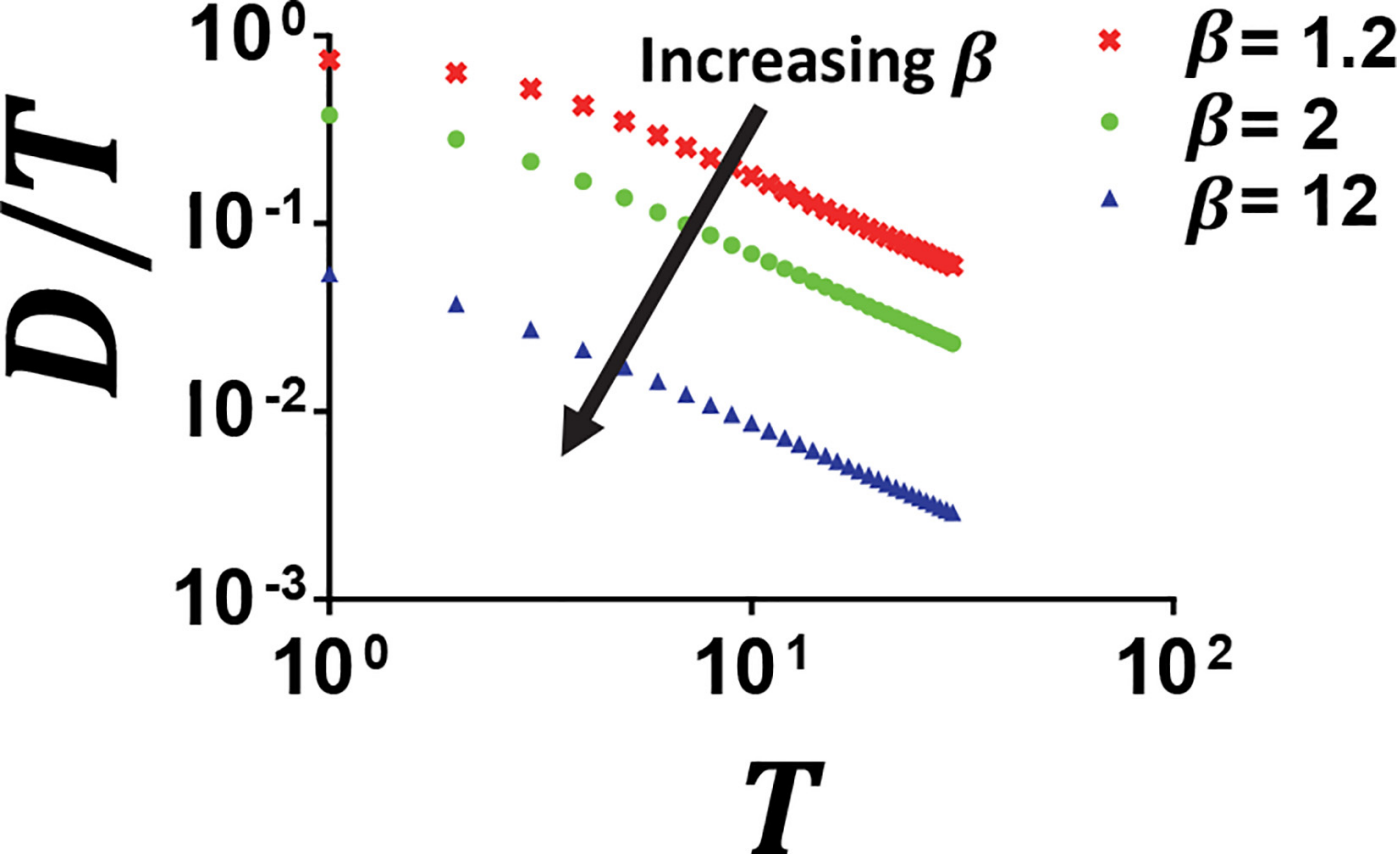
Dynamic constraint underlying counting capability. The analytical border $\frac{1-e^{-D}}{1-e^{-T}}=\frac{1}{\beta }$ describes the effect of changing ***β*** (*β* = 12 or 2 or 1.2) on the relative duration (***D***/***T***) at which counting is lost. To the left of the solution for each ***β*** is the region with counting while the region to the right has no counting capability.

Fig 5 shows that the duration at which counting exists decreases with increasing ***β***. Consistent with the numerical analysis where slopes are generally higher at shorter pulse durations with an increasing ***β***, higher synthesis rates of ***X*** shorten the duration range for which counting can occur (Fig 4). Thus, a higher ***β*** means that ***X*** will reach the threshold at shorter pulse durations, resulting in the loss of the counting capability beyond this duration. The curve of the boundary signifies that increasing the cycle period has little impact on the duration at which counting is lost (Fig 5). More specifically, at the analytical border, the duration at which $\frac{1-e^{-D}}{1-e^{-T}}=\frac{1}{\beta }$ is maintained relies on ***β***. However, at low periods, the duration at which counting exists relies more heavily on the cycle period. As the pulse duration approaches the cycle period, there is less time for ***X*** to return to the basal value before subsequent pulses. This effect has a greater impact in the case of low cycle periods. Although the basal level of ***X*** will never return to the initial value, if ***x*** remains below the activation threshold, negligible increases can maintain the counting capability.

The analytical solution **1** > ***β***(**1** − ***e***^−***D***^) defines the conditions under which indefinite counting occurs based on the peak concentration of the intermediate. When ***γ***_***o***_ > **0**, however, the number of pulses any particular system can count becomes finite due to the saturation of the reporter. This is described in S1 Text, which gives the final level of ***R*** at time ***τ***_***E***_ (> (***k*** − **1**)***T*** + ***D*** + ***τ***_**0**_) after any arbitrary number of pulses (***k*** > **0**) when **1** > ***β***(**1** − ***e***^−***D***^) as $r(\tau_{E})=\frac{\beta }{\gamma_{o}}$(**1**-$e^{-\gamma_{o}D}$)$\sum_{i=0}^{k-1}e^{-\gamma_{o}(\tau_{E}-\tau_{0}-(iT+D))}$. ***τ***_**0**_, the time at which the pulses start, can impact the parameter range with the counting capability. Depending on ***γ***_***o***_, a series of pulses that start at the beginning of the simulation interval can have a different ***r***(***τ***_***E***_) than those that start near the end of the interval. To analyze the effect of the other parameters, we constrain our analysis by assuming a constant ***τ***_**0**_ for all simulations. Here, ***r***(***τ***_***E***_) is a monotonically increasing function of the number of pulses, regardless of the other parameters (as long as ***X*** does not accumulate beyond its threshold to trigger the degradation of ***R***). When ***γ***_***o***_ **= 0**, ***r***(***τ***_***E***_) is proportional to ***k***: ***r***(***τ***_***E***_) = ***kβ***. However, the dependence of ***r***(***τ***_***E***_) on ***k*** deviates from the proportionality as ***γ***_***o***_ increases. This is consistent with the numerical simulation results illustrated by Fig 3.

The key constraint for IFFLs to distinguish oscillatory signals resides in its ability for temporal adaptation where the network responds with a pulse to a sustained input. As long as an IFFL maintains this characteristic, the motif can respond to periodic stimulation and act as a pulse counter. Each time the input is removed, the intermediate is reset and the system can respond to subsequent pulses. We illustrate this principle by considering alternative implementations of the IFFL satisfying this constraint.

We examine the reliance of counting on the current architecture by shifting the ultrasensitive response to the production of ***X*** through a positive feedback loop (S1 Text). Like the initial model, this alternative model maintains the ability to count in the base case with no endogenous ***R*** degradation (S3 Fig). Here, when ***β*** is large, the linear dependence of the degradation of ***R*** on both ***X*** and ***R*** causes any ***X*** to induce the degradation of ***R***, preventing counting. However, when ***β*** is small, ***R*** degrades slowly because of low concentrations of ***X***, preventing the differentiation between sustained and pulsing inputs. Like in the initial model, ***γ***_***o***_ dictates the length of time during which ***R*** remains constant and should be minimized to maximize the duration range in which counting can occur. For a large ***γ***_***R***_, the presence of a small concentration of ***R*** or ***X*** will lead to the efficient degradation of ***R***, preventing the ability to count. Meanwhile, a small ***γ***_***R***_ will be unable to induce the effective degradation of ***R*** even at high concentrations of ***X***. To demonstrate the robustness of this counting mechanism, we develop additional models to take into account common properties of biological systems including time delay, modifications to the current architecture, and noise (S1 Text; S1, S3, S5, S8 and S9 Figs).

## Discussion

Here, we analyze an underappreciated phenomenon in information processing by signaling networks: processing of oscillatory signals. In this context, we illustrate the unique ability of incoherent feedforward loops (IFFL) with proper network parameters to distinguish transient (or oscillatory) and sustained signals. For oscillatory signals, we analyze the extent to which the network is able to count the number of pulses. Additionally, we establish the dynamic constraints of the input signals and network parameters such that counting can occur. With the appropriate parameters, the ability of an IFFL motif to respond to transient signals but not to sustained signals relies upon the property of temporal adaptation in response to a sustained signal. Insights from our model can be used to identify natural information decoding systems and understand the effect of perturbations to such networks.

Our results can be applied to GtaC shuttling in social amoeba, which differentiates between pulsing and sustained cAMP and matches optimal signals to the ideal transcript accumulation. Cai et. al. show that when the network regulating CsaA is subjected to high frequency inputs, GtaC is unable to respond to each pulse, resulting in a decreased transcript accumulation. Similarly, a lower than optimal (natural) cAMP frequency results in a decrease in the overall transcript concentration. This phenomenon suggests the presence of a band-pass filter. As in Fig 5, this system illustrates the idea that a decreased frequency (higher period) corresponds to a reduced fraction of the period during which counting can occur. Here, the parameters of the endogenous circuit dictate the range of frequencies that can be decoded resulting in dynamics similar to those explained by our IFFL model.

In a similar manner, the frequency of ERK activation has been implicated in cell-fate determination. While previous studies have demonstrated the dependence of differential cell fates (differentiation or proliferation) on specific growth factors, Ryu et. al. illustrate that this decision relies more heavily on the frequency of ERK activity than on the growth-factor identity [49]. Unlike with the sustained application of growth factors, synthetic pulses provide control over the frequency of the ERK activity. This results in differentiation at intermediate frequencies and proliferation at high and low frequencies with a growth factor traditionally known to solely induce proliferation. While this shift in cell fates has been shown experimentally, the mechanisms underlying this frequency decoding are still unknown.

Evolution may have selected for an optimal pulse frequency for social amoeba and in mammalian cell differentiation for different purposes. However, despite differences in signaling dynamics, the IFFL is a general motif found downstream of divergent signals in both cases. Generally, IFFLs are known for acting as sign-sensitive accelerators by increasing the response time of gene expression, dictated by the specific combination of kinetic parameters [13]. While some parameter sets allow the IFFL to act as pulse generators or band-pass filters, others speed up an output response [13, 50]. In addition, the IFFL has been shown to generate both time-dependent and dose-dependent biphasic responses [51]. Our analysis defines the quantitative constraints of another property of IFFLs, the capability for counting.

In natural systems, motifs do not exist in isolation and the dynamics of a motif in isolation may not persist when placed in the context of a larger network. Despite this caveat, our analysis is valuable for two major reasons. First, we identify a new property of IFFLs—the potential for information processing. The importance of IFFLs in signal processing is implied with its presence in many natural networks that are able to respond differentially to oscillatory and sustained signals (Fig 1). Considering this caveat, we explore the extent by which the counting mechanism, can persist in the presence of different perturbations in S1 Text (e.g. introduction of time delay, alterations to the current architecture, and cellular noise). Second, our analysis is useful for guiding the design and implementation of synthetic gene circuits, which are often intended to operate in relative isolation from other regulatory networks in the host cell.

Understanding the constraints of this network for decoding mechanisms is important in the design of synthetic circuits with more complex functions [52, 53]. The conclusions from our study can be used in the design of a synthetic pulse counter [54, 55]. Many synthetic gene circuits respond to the characteristics of static signals. In contrast, circuits adapted from our model would respond to the temporal dynamics of input signals. This would provide a wider range of input attributes, giving rise to a larger spectrum of potential circuit responses. In addition, by matching IFFL characteristics to natural cellular oscillations, synthetic signal decoding circuits would have the capability to respond to natural as well as synthetic stimuli.

Natural systems including those involved in cell proliferation, cell death, and neural regeneration convert oscillatory and sustained signals into distinct biological outcomes. In the case of disease, these circuits become deregulated, altering the function of networks required for information encoding or decoding. Insights derived from our model for the pulse counting mechanism will be important for future work in understanding the ways in which these perturbations impact the way cells decode information.

## Methods

We use the following system of ODEs to define the IFFL network:

$$\frac{d[R]}{dt}=k_{1}\;\Phi (t)-d_{R}\;\frac{{\left[X\right]}^{n}}{{\left[X\right]}^{n}+K_{x}^{n}}\;[R]-d_{Ro}[R]$$ (5)

$$\frac{d[X]}{dt}=k_{2}\Phi (t)-d_{x}[X]$$ (6)

where ***k***_**1**_ and ***k***_**2**_ are rate constants for the production of ***R*** and ***X*** respectively, and ***d***_***x***_ and ***d***_***Ro***_ are rate constants for the endogenous degradation of ***X*** and ***R*** respectively. The term $\frac{{\left[X\right]}^{n}}{{\left[X\right]}^{n}+K_{x}^{n}}$ represents the threshold response through Hill kinetics for the induction of the degradation of ***R*** by ***X***, with a maximal degradation rate of ***d***_***R***_. Unless noted otherwise, we define ***Φ***(***t***) as a periodic square wave function with *k* pulses: ***Φ***(***t***) = **1** when ***iT***′ + ***t***_***o***_ ≤ ***t*** < ***iT***′ + ***D***′ + ***t***_***o***_ and ***Φ***(***t***) = **0** otherwise. Here ***i*** (**0** ≤ ***i*** < ***k***) is the pulse index, ***t***_***o***_ is the start time, ***T***′ is the cycle period, and ***D***′ is the pulse duration.

We assume a square waveform input to simplify analysis. However, this assumption can be relaxed; numerical simulations indicate that other wave forms, such as sinusoidal input signals (S1 Fig), can also be counted. In our model, we assume first-order kinetics for the endogenous degradation of ***R*** and ***X***. We assume that the induced degradation by ***X*** occurs through an ultrasensitive threshold response. In general, this arm can be found in other positions while maintaining the network structure. Our analysis can be generalized to other forms of the motif, as illustrated by the alternative models (S1 Text).

We non-dimensionalize the model by defining:

$$x=\frac{\left[X\right]}{K_{x}},\;r=\frac{{[R]k}_{2}}{k_{1}K_{x}},\;\tau =td_{x},\;\tau_{0}=t_{0}d_{x},\;D=D\prime {\;d}_{x},\;T=T\prime {\;d}_{x},\;\gamma_{R}=\frac{d_{R}}{d_{x}},\;\gamma_{o}=\frac{d_{Ro}}{d_{x}},\;and\;\beta =\frac{k_{2}}{K_{x}d_{x}}$$

This leads to Eqs 1 and 2.

## Supporting Information

S1 Text Supporting Information. (DOCX)

S1 Fig Incoherent Feedforward Loop and pulse counting. (A) Time courses for pulses defined by a sine function. This panel uses *β* = 1.2, *γ*_*R*_ = 10, *γ*_*O*_ = 0, and *T* = 4 (Left: *D* = 1 Right: *D* = 3). The top row contains the time courses of the input pulses for two different pulse durations, either a pulsing input or a simulated sustained input (with a sine waveform). The second row shows the time courses for *X*. The bottom row shows the time courses for *R*. With identical parameters, an IFFL motif can generate two distinct outputs depending on the length of the duration of the input pulses. When *X* is below the threshold of induction, the circuit maintains the ability to produce a stepwise increase of R. However, when *X* overcomes the threshold, the circuit loses this ability. For a single parameter set, both outputs are desired to quantify the system as being capable of counting. (B) Calibration curve for ideal counting. This panel uses *β* = 1.2, *γ*_*R*_ = 10, *γ*_*O*_ = 0, and *T* = 4 (*D* = 1). The sample calibration curve is for a pulse duration within the optimal duration range, therefore it is able to demonstrate ideal counting. With an increasing number of input pulses from 1–4 in the top panel, *R* exhibits a stepwise increase. The linearity is demonstrated in the bottom panel by R^2^ > 0.99.
(TIF)

S2 Fig Failure in counting. Here, A and B both use *β* = 1.2, *γ*_*R*_ = 10, *γ*_*O*_ = 0, and *T* = 4 (*D* = 3), a parameter set which demonstrates the inability to count. (A) Time course for failed counting. With an increasing number of pulses, *X* overcomes the activation threshold and the ability to produce a stepwise increase of *R* is lost. (B) Calibration curve for failed counting. The sample calibration curve is for a pulse duration outside the optimal duration range, therefore it is able to demonstrate the case when counting fails. With an increasing number of input pulses from 1–10 *r* is suppressed and the non-linearity is demonstrated by R^2^ < 0.99.
(TIF)

S3 Fig Counting with the alternative model. (A) Alternative incoherent feedforward loop motif (Model in S1). In this model, a pulsing input simultaneously stimulates the production of *X* and *R*. Here, the threshold response is implemented with the additional production of *X* through a positive feedback loop. (B) Calibration curves for ideal or failed counting. The top panel uses *β* = 1.2, *γ*_*R*_ = 0.01, *γ*_*O*_ = 0, *α* = 100, and *T* = 10 (*D* = 1). The sample calibration curve is for a pulse duration within the optimal duration range, therefore it demonstrates counting. The bottom panel uses *β* = 1.2, *γ*_*R*_ = 0.01, *γ*_*O*_ = 0, *α* = 100, and *T* = 10 (*D* = 9). The sample calibration curve is for a pulse duration outside the optimal duration range, therefore it demonstrates the case when counting fails.
(TIF)

S4 Fig An IFFL can maintain robust counting of oscillating signals in the presence of additional time delay in the inhibition arm. (A) Signal processing by an IFFL (Model in S2). In this model, a pulsing input (*S*) simultaneously stimulates the production of an intermediate (*X*_1_) and a reporter (*R*). The first intermediate (*X*_1_) then activates the production of the second intermediate (*X*) that induces the degradation of *R* through a threshold response. The fundamental constraints for counting shown are based on the full model (S1 Text). (B) Time courses demonstrate counting mechanism. Using *β*′ = 1, *β*″ = 1.2, *γ*_*R*_ = 10, *δ* = 1, *γ*_*O*_ = 0, and *T* = 4 (Left: *D* = 1 Right: *D* = 3). The top row contains time courses of the input pulses for two different pulse durations, either a pulsing input or a simulated sustained input. The second row shows time courses for *X*_1_. The third row shows time courses for *X*. The bottom row shows time courses for *R*. Despite the addition of time delay (of ~10% of the pulse duration) through an additional component, the IFFL motif maintains the ability to distinguish input signals with longer or shorter pulses.
(TIF)

S5 Fig An IFFL can maintain robust counting of oscillating signals in the presence of additional time delay in the activation arm. (A) Signal processing by an IFFL (Model in S3). In this model, a pulsing input (*S*) simultaneously stimulates the production of an intermediate (*X*) and an intermediate reporter (*R*_1)_. The intermediate reporter (*R*_1_) then activates the production of the reporter (*R*), which is degraded by the intermediate (*X*) through a threshold response. The fundamental constraints for counting shown are based on the full model (S1 Text). (B) Time courses demonstrate counting mechanism. Using *β*′ = 5, *β*″ = 1.2, *γ*_*R*_ = 10, *γ*_*R*1_ = 5, *γ*_*O*_ = 0, and *T* = 4 (Left: *D* = 1 Right: *D* = 3). The top row contains time courses of the input pulses for two different pulse durations, either a pulsing input or a simulated sustained input. The second row shows time courses for *X*. The third row shows time courses for *R*_1_. The bottom row shows time courses for *R*. Despite the addition of time delay (of ~10% of the pulse duration) through an additional component, the IFFL motif maintains the ability to distinguish input signals with longer or shorter pulses.
(TIF)

S6 Fig Distinguishing between a sustained input and an oscillatory input that are on average at the same level. (A) Time courses demonstrate counting mechanism. Using *β* = 1.2, *γ*_*R*_ = 10, *γ*_*O*_ = 0, and *T* = 4 (Left: *D* = 1 Right: *D* = 3). The top row contains time courses of the input pulses for two different pulse durations, either a pulsing input or a simulated sustained input. The second row shows time courses for *X*. The bottom row shows time courses for *R*. (B) Time courses demonstrate differentiation between a sustained and oscillatory input. Using *β* = 0.337, *γ*_*R*_ = 10, *γ*_*O*_ = 0. Here, the amplitude of the sustained input is equivalent to the mean of the oscillating input in panel A (*D* = 1).
(TIF)

S7 Fig Ultrasensitivity (as indicated by a high Hill coefficient) in induced degradation is critical for robust counting. Time courses of the reporter. Using *β* = 1.2, *γ*_*R*_ = 10, *γ*_*O*_ = 0, *T* = 4, *D* = 1. Here, we show time courses for *R* with varying values for the Hill coefficient (n = 5, 50, 110, or 1000) to demonstrate that the specific value for n is irrelevant. The hill coefficient must simply be high enough to induce a threshold response.
(TIF)

S8 Fig Stochastic simulations of IFFL. (A) Time courses demonstrate the effect of noise in the case of a small molecular number (Model S5). Using *k*_1_ = *k*_2_ = 5, *d*_*x*_ = 1, *d*_*R*_ = 10, n = 100, *d*_*Ro*_ = 0, *K*_*x*_ = 4, and *T* = 4 (*D* = 1) in equations 20–21. The top row shows time courses of the input pulses in the case of an oscillating input. The second row shows time courses for *R* in the absence of noise. The third row shows time courses for *X* in the presence of noise (*ξ* = 10). The fourth row shows time courses for *R* in the presence of noise. The bottom row shows the calibration curve for the system in the presence of noise. With an increasing number of input pulses from 1–10, *R* is unable to exhibit a stepwise increase due to the impact of noise on the low molecular number of the components; this poor linearity is demonstrated by R^2^ < 0.99. (B) Time courses demonstrate the effect of noise in the case of an increasing molecular number (Model S5). Using *k*_1_ = *k*_2_ = 50, *d*_*x*_ = 1, *d*_*R*_ = 10, n = 100, *d*_*Ro*_ = 0, *K*_*x*_ = 40, and *T* = 4 (*D* = 1) in equations 20–21. The top row contains time courses of the input pulses in the case of an oscillating input. The second row shows time courses for *R* in the absence of noise. The third row shows time courses for *X* in the presence of noise (*ξ* = 10). The fourth row shows time courses for *R* in the presence of noise. The bottom row shows the calibration curve for the system in the presence of noise. With an increasing number of input pulses from 1–10, *R* is unable to exhibit a stepwise increase due to the impact of noise on the low molecular number of the components; the poor linearity is demonstrated by R^2^ < 0.99. (C) Time courses demonstrate the effect of noise in the case of a sufficiently high molecular number (Model S5). Using *k*_1_ = *k*_2_ = 500, *d*_*x*_ = 1, *d*_*R*_ = 10, n = 100, *d*_*Ro*_ = 0, *K*_*x*_ = 400, and *T* = 4 (*D* = 1) in equations 20–21. The top row contains time courses of the input pulses in the case of an oscillating input. The second row shows time courses for *R* in the absence of noise. The third row shows time courses for *X* in the presence of noise (*ξ* = 10). The fourth row shows time courses for *R* in the presence of noise. The bottom row shows the calibration curve for the system in the presence of noise. With an increasing number of input pulses from 1–10, *R* is able to exhibit a stepwise increase despite the presence of noise due to the sufficiently high molecular number. Here, the linearity is demonstrated by R^2^ ≥ 0.99.
(TIF)

S9 Fig The implementation of the threshold response in an IFFL through repression. (A) Signal processing by an IFFL (Model S6). In this model, a pulsing input (*S*) simultaneously stimulates the production of an intermediate (*X*) and a reporter (*R*). The intermediate represses the production of *R* through a threshold response. The fundamental constraints for counting shown are based on the model described in S1 Text. (B) Time courses demonstrate counting mechanism. Using *β* = 12, *γ*_*O*_ = 0, and *T* = 4 (Left: *D* = 1 Right: *D* = 3). The top row contains time courses of the input pulses for two different pulse durations, either a pulsing input or a simulated sustained input. The second row shows time courses for *X*. The bottom row shows time courses for *R*. Here, an IFFL motif can generate two distinct outputs depending on the length of the duration of the input pulses.
(TIF)
