# Supplementary material for: Processing Oscillatory Signals by Incoherent Feedforward Loops
Source: PLoS Comput Biol. 2016 Sep 13;12(9):e1005101. doi: 10.1371/journal.pcbi.1005101 (PMC5021367; doi:10.1371/journal.pcbi.1005101)
Supplement: S1 Text — (DOCX) [file pcbi.1005101.s001.docx]

**Processing oscillatory signals by incoherent feedforward loops**

Carolyn Zhang^1^, Ryan Tsoi^1+^, Feilun Wu^1+^, Lingchong You^1, 2, 3^*

^1^Department of Biomedical Engineering, Duke University, Durham, North Carolina, 27708, USA

^2^Center for Genomic and Computational Biology, Duke University, Durham, North Carolina, 27708, USA

^3^Department of Molecular Genetics and Microbiology, Duke University School of Medicine, North Carolina, 27708, USA

^+^These authors contributed equally

*Corresponding author. Department of Biomedical Engineering, Duke University, CIEMAS 2355, 101 Science Drive, Box 3382, Durham, North Carolina, 27708, USA

Tel.: +1 (919)660-8408; Fax: +1 (919)668-0795; E-mail:you@duke.edu

**S1 Text. Model Development**

**S1. Alternative model: positive feedback on X synthesis**

This alternative model consists of three components, an input node ($\boldsymbol{S}$), an intermediate node ($\boldsymbol{X}$), and an output node ($\boldsymbol{R}$). In contrast to the original model, $\boldsymbol{X}$ is synthesized through an additional positive feedback regulatory loop to produce an ultrasensitive response. We use the following system of ODEs to define the network:

$\frac{\boldsymbol{d[R]}}{\boldsymbol{dt}}\boldsymbol{=}\boldsymbol{k}_{\boldsymbol{1}}\boldsymbol{\Phi(t)-}\boldsymbol{d}_{\boldsymbol{R}}\boldsymbol{[X]}\left[ \boldsymbol{R} \right]\boldsymbol{-}\boldsymbol{d}_{\boldsymbol{Ro}}\boldsymbol{[R]}$**, (1)**

$\frac{\boldsymbol{d[X]}}{\boldsymbol{dt}}\boldsymbol{=}\boldsymbol{k}_{\boldsymbol{2}}\boldsymbol{\Phi(t)+}\boldsymbol{k}_{\boldsymbol{3}}\frac{\left[ \boldsymbol{X} \right]^{\boldsymbol{n}}}{\left[ \boldsymbol{X} \right]^{\boldsymbol{n}}\boldsymbol{+}\boldsymbol{K}_{\boldsymbol{x}}^{\boldsymbol{n}}}\boldsymbol{-}\boldsymbol{d}_{\boldsymbol{x}}\boldsymbol{[X]}$**, (2)**

where $\boldsymbol{k}_{\boldsymbol{1}}$**,**  $\boldsymbol{k}_{\boldsymbol{2}}$**,** and $\boldsymbol{k}_{\boldsymbol{3}}$ are the rate constants for the production of $\boldsymbol{R}$, $\boldsymbol{X}$, and the positive feedback loop of $\boldsymbol{X}$ respectively, and $\boldsymbol{d}_{\boldsymbol{x}}$ and $\boldsymbol{d}_{\boldsymbol{Ro}}$ are the rate constants for the endogenous degradation of $\boldsymbol{X}$ and $\boldsymbol{R}$ respectively. Additionally, the maximal degradation rate of $\boldsymbol{R}$ by $\boldsymbol{X}$ is $\boldsymbol{d}_{\boldsymbol{R}}$. The term $\frac{\left[ \boldsymbol{X} \right]^{\boldsymbol{n}}}{\left[ \boldsymbol{X} \right]^{\boldsymbol{n}}\boldsymbol{+}\boldsymbol{K}_{\boldsymbol{x}}^{\boldsymbol{n}}}$ represents the ultrasensitive response through Michaelis-Menten kinetics for the production of $\boldsymbol{X}$ (positive feedback loop) of strength $\boldsymbol{k}_{\boldsymbol{3}}$. $\boldsymbol{\Phi(t)}$ defines the shape of the input function (see main text)**.**

We non-dimensionalize the model by defining:

$\boldsymbol{x=}\frac{\boldsymbol{[X]}}{\boldsymbol{K}_{\boldsymbol{x}}}$, $\boldsymbol{r=}\frac{\boldsymbol{[R]k}_{\boldsymbol{2}}}{\boldsymbol{k}_{\boldsymbol{1}}\boldsymbol{K}_{\boldsymbol{X}}}$, $\boldsymbol{\tau=t}\boldsymbol{d}_{\boldsymbol{x}}$, $\boldsymbol{\tau}_{\boldsymbol{0}}\boldsymbol{=}\boldsymbol{t}_{\boldsymbol{0}}\boldsymbol{d}_{\boldsymbol{x}}\mathbf{,}\boldsymbol{\gamma}_{\boldsymbol{R}}\boldsymbol{=}\frac{{\boldsymbol{K}_{\boldsymbol{x}}\boldsymbol{d}}_{\boldsymbol{R}}}{\boldsymbol{d}_{\boldsymbol{x}}}$, $\boldsymbol{\gamma}_{\boldsymbol{o}}\boldsymbol{=}\frac{\boldsymbol{d}_{\boldsymbol{Ro}}}{\boldsymbol{d}_{\boldsymbol{x}}}$, $\boldsymbol{\alpha}\boldsymbol{=}\frac{\boldsymbol{k}_{\boldsymbol{3}}}{\boldsymbol{K}_{\boldsymbol{x}}\boldsymbol{d}_{\boldsymbol{x}}}$ and $\boldsymbol{\beta=}\frac{\boldsymbol{k}_{\boldsymbol{2}}}{\boldsymbol{K}_{\boldsymbol{x}}\boldsymbol{d}_{\boldsymbol{x}}}$

This leads to:

$\frac{\boldsymbol{dr}}{\boldsymbol{d\tau}}\boldsymbol{=\beta\Phi(}\mathsf{\tau)} \boldsymbol{-}\boldsymbol{(\gamma}_{\boldsymbol{R}}\boldsymbol{x+}\boldsymbol{\gamma}_{\boldsymbol{o}}\boldsymbol{)r}$**, (3)**

$\frac{\boldsymbol{dx}}{\boldsymbol{d\tau}}\boldsymbol{=\beta\Phi(}\mathsf{\tau)} \boldsymbol{+ \alpha}\frac{\boldsymbol{x}^{\boldsymbol{n}}}{\boldsymbol{x}^{\boldsymbol{n}}\boldsymbol{+1}}\boldsymbol{-x}$ **, (4)**

where ***x*** , ***r*** , and $\boldsymbol{\tau}$ are dimensionless variables such that $\boldsymbol{x}$ is the intermediate, $\boldsymbol{r}$ is the output, and $\boldsymbol{\tau}$ is time. $\boldsymbol{\gamma}_{\boldsymbol{R}}$, $\boldsymbol{\gamma}_{\boldsymbol{o}}$, $\boldsymbol{\beta}$, and $\boldsymbol{\alpha}$ are dimensionless parameters such that $\boldsymbol{\gamma}_{\boldsymbol{o}}$ is the ratio of the endogenous degradation rate of $\boldsymbol{R}$ to the endogenous degradation rate of $\boldsymbol{X}$, $\boldsymbol{\gamma}_{\boldsymbol{R}}$ is the relative induced degradation rate of $\boldsymbol{R}$ by $\boldsymbol{X}$, $\boldsymbol{\beta}$ is the relative production rate of $\boldsymbol{X}$ and $\boldsymbol{R}$, and $\boldsymbol{\alpha}$ is the relative strength of the positive feedback.

**S2. Alternative model: time delay in the inhibition arm of IFFL.**

Here, we incorporate time delay into the inhibition arm of the IFFL through the addition of a second intermediate component $\boldsymbol{X}_{\boldsymbol{1}}$. We use the following system of ODEs to define the network:

$\frac{\boldsymbol{d[R]}}{\boldsymbol{dt}}\boldsymbol{=}\boldsymbol{k}_{\boldsymbol{1}}\boldsymbol{\Phi(t)} \boldsymbol{-}\boldsymbol{d}_{\boldsymbol{R}} \frac{\left[ \boldsymbol{X} \right]^{\boldsymbol{n}}}{\left[ \boldsymbol{X} \right]^{\boldsymbol{n}}\boldsymbol{+}\boldsymbol{K}_{\boldsymbol{x}}^{\boldsymbol{n}}} \left[ \boldsymbol{R} \right]\boldsymbol{-}\boldsymbol{d}_{\boldsymbol{Ro}}\boldsymbol{[R]}$ **, (5)**

$\frac{\boldsymbol{d[X]}}{\boldsymbol{dt}}\boldsymbol{=}\boldsymbol{k}_{\boldsymbol{2}}\boldsymbol{[}\boldsymbol{X}_{\boldsymbol{1}}\boldsymbol{]-}\boldsymbol{d}_{\boldsymbol{x}}\boldsymbol{[X]}$ **, (6)**

$\frac{\boldsymbol{d[}\boldsymbol{X}_{\boldsymbol{1}}\boldsymbol{]}}{\boldsymbol{dt}}\boldsymbol{=}\boldsymbol{k}_{\boldsymbol{3}}\boldsymbol{\Phi(t)} \boldsymbol{-}\boldsymbol{d}_{\boldsymbol{x}\boldsymbol{1}}\boldsymbol{[}\boldsymbol{X}_{\boldsymbol{1}}\boldsymbol{]}$ **, (7)**

where $\boldsymbol{k}_{\boldsymbol{1}}$, $\boldsymbol{k}_{\boldsymbol{2}}$, and $\boldsymbol{k}_{\boldsymbol{3}}$ are rate constants for the production of $\boldsymbol{R}$, $\boldsymbol{X}$, and $\boldsymbol{X}_{\boldsymbol{1}}$ respectively, and $\boldsymbol{d}_{\boldsymbol{x}}$, $\boldsymbol{d}_{\boldsymbol{x}\boldsymbol{1}}$, and $\boldsymbol{d}_{\boldsymbol{Ro}}$ are rate constants for the endogenous degradation of $\boldsymbol{X}$, $\boldsymbol{X}_{\boldsymbol{1}}$, and $\boldsymbol{R}$ respectively. The term $\frac{\left[ \boldsymbol{X} \right]^{\boldsymbol{n}}}{\left[ \boldsymbol{X} \right]^{\boldsymbol{n}}\boldsymbol{+}\boldsymbol{K}_{\boldsymbol{x}}^{\boldsymbol{n}}}$ represents the threshold response through Hill kinetics for the induction of the degradation of $\boldsymbol{R}$ by $\boldsymbol{X}$, with a maximal degradation rate of $\boldsymbol{d}_{\boldsymbol{R}}$. $\boldsymbol{\Phi(t)}$ defines the shape of the input function (see main text)**.**

We non-dimensionalize the model by defining:

$\boldsymbol{x=}\frac{\boldsymbol{[X]}}{\boldsymbol{K}_{\boldsymbol{x}}}$**,** $\boldsymbol{x}_{\boldsymbol{1}}\boldsymbol{=}\frac{\boldsymbol{[}\boldsymbol{X}_{\boldsymbol{1}}\boldsymbol{]}}{\boldsymbol{K}_{\boldsymbol{x}}}$**,** $\boldsymbol{r=}\frac{\boldsymbol{[R]k}_{\boldsymbol{3}}}{\boldsymbol{k}_{\boldsymbol{1}}\boldsymbol{K}_{\boldsymbol{x}}\boldsymbol{\delta}}$**,** $\boldsymbol{\delta=}\frac{\boldsymbol{d}_{\boldsymbol{x}\boldsymbol{1}}}{\boldsymbol{d}_{\boldsymbol{x}}}$**,** $\boldsymbol{\tau=t}\boldsymbol{d}_{\boldsymbol{x}}$**,** $\boldsymbol{\tau}_{\boldsymbol{0}}\boldsymbol{=}\boldsymbol{t}_{\boldsymbol{0}}\boldsymbol{d}_{\boldsymbol{x}}$**,** $\boldsymbol{\gamma}_{\boldsymbol{R}}\boldsymbol{=}\frac{\boldsymbol{d}_{\boldsymbol{R}}}{\boldsymbol{d}_{\boldsymbol{x}}}$**,** $\boldsymbol{\gamma}_{\boldsymbol{o}}\boldsymbol{=}\frac{\boldsymbol{d}_{\boldsymbol{Ro}}}{\boldsymbol{d}_{\boldsymbol{x}}}$**,** $\boldsymbol{\beta'=}\frac{\boldsymbol{k}_{\boldsymbol{2}}}{\boldsymbol{d}_{\boldsymbol{x}}}$ **,** and $\boldsymbol{\beta''=}\frac{\boldsymbol{k}_{\boldsymbol{3}}}{{\boldsymbol{K}_{\boldsymbol{x}}\boldsymbol{d}}_{\boldsymbol{x}\boldsymbol{1}}}$

This leads to the model equations:

$\frac{\boldsymbol{dr}}{\boldsymbol{d\tau}}\boldsymbol{=\beta''\Phi(}\mathsf{\tau)-}\boldsymbol{(\gamma}_{\boldsymbol{R}}\frac{\boldsymbol{x}^{\boldsymbol{n}}}{\boldsymbol{x}^{\boldsymbol{n}}\boldsymbol{+1}}\boldsymbol{+}\boldsymbol{\gamma}_{\boldsymbol{o}}\boldsymbol{)r}$ **, (8)**

$\frac{\boldsymbol{d}\boldsymbol{x}_{\boldsymbol{1}}}{\boldsymbol{d\tau}}\boldsymbol{=}\boldsymbol{\delta(\beta}^{\boldsymbol{''}}\boldsymbol{\Phi(}\mathsf{\tau)-}\boldsymbol{x}_{\boldsymbol{1}}\boldsymbol{)}$ **, (9)**

$\frac{\boldsymbol{dx}}{\boldsymbol{d\tau}}\boldsymbol{=\beta'}\boldsymbol{x}_{\boldsymbol{1}}\boldsymbol{-x}$ **, (10)**

As the length of the time delay of the inhibition arm increases, the ability of $\boldsymbol{X}$ to respond to each pulse is delayed, which limits its capability to induce the degradation of $\boldsymbol{R}$. Increasing time delay in this manner will not have a drastic impact on counting since its main effect is on the reduction of the activity of $\boldsymbol{X}$ (a cascade acts as a low pass filter). However, it would reduce the ability of the IFFL to differentiate between transient and sustained signals. As a result, as $\boldsymbol{\delta}$ decreases, the range of parameters which are capable of differentiating between transient and sustained signals decreases. We demonstrate in S4 Fig that the addition of a sufficiently short time delay has little impact on the general properties dictating both the counting capability and the ability to differentiate between the different types of signals.

**S3.** **Alternative model: time delay in the activation arm of IFFL.**

Alternatively, the time delay can be incorporated into the activation arm of the IFFL through the addition of an intermediate component $\boldsymbol{R}_{\boldsymbol{1}}$. We use the following system of ODEs to define the network:

$\frac{\boldsymbol{d[R]}}{\boldsymbol{dt}}\boldsymbol{=}\boldsymbol{k}_{\boldsymbol{1}}\boldsymbol{[}\boldsymbol{R}_{\boldsymbol{1}}\boldsymbol{]-}\boldsymbol{d}_{\boldsymbol{R}} \frac{\left[ \boldsymbol{X} \right]^{\boldsymbol{n}}}{\left[ \boldsymbol{X} \right]^{\boldsymbol{n}}\boldsymbol{+}\boldsymbol{K}_{\boldsymbol{x}}^{\boldsymbol{n}}} \left[ \boldsymbol{R} \right]\boldsymbol{-}\boldsymbol{d}_{\boldsymbol{Ro}}\boldsymbol{[R]}$ **, (11)**

$\frac{\boldsymbol{d[}\boldsymbol{R}_{\boldsymbol{1}}\boldsymbol{]}}{\boldsymbol{dt}}\boldsymbol{=}\boldsymbol{k}_{\boldsymbol{2}}\boldsymbol{\Phi}\left( \boldsymbol{t} \right)\boldsymbol{-}\boldsymbol{d}_{\boldsymbol{R}\boldsymbol{1}}\boldsymbol{[}\boldsymbol{R}_{\boldsymbol{1}}\boldsymbol{]}$ **, (12)**

$\frac{\boldsymbol{d[X]}}{\boldsymbol{dt}}\boldsymbol{=}\boldsymbol{k}_{\boldsymbol{3}}\boldsymbol{\Phi}\left( \boldsymbol{t} \right)\boldsymbol{-}\boldsymbol{d}_{\boldsymbol{x}}\boldsymbol{[X]}$ **, (13)**

where $\boldsymbol{k}_{\boldsymbol{1}}$, $\boldsymbol{k}_{\boldsymbol{2}}$, and $\boldsymbol{k}_{\boldsymbol{3}}$ are the rate constants for the production of $\boldsymbol{R}$, $\boldsymbol{R}_{\boldsymbol{1}}$, and $\boldsymbol{X}$ respectively, and $\boldsymbol{d}_{\boldsymbol{x}}$, $\boldsymbol{d}_{\boldsymbol{R}\boldsymbol{1}}$, and $\boldsymbol{d}_{\boldsymbol{Ro}}$ are the rate constants for the endogenous degradation of $\boldsymbol{X}$, $\boldsymbol{R}_{\boldsymbol{1}}$, and $\boldsymbol{R}$ respectively. The term $\frac{\left[ \boldsymbol{X} \right]^{\boldsymbol{n}}}{\left[ \boldsymbol{X} \right]^{\boldsymbol{n}}\boldsymbol{+}\boldsymbol{K}_{\boldsymbol{x}}^{\boldsymbol{n}}}$ represents the threshold response through Hill kinetics for the induction of the degradation of $\boldsymbol{R}$ by $\boldsymbol{X}$, with a maximal degradation rate of $\boldsymbol{d}_{\boldsymbol{R}}$. $\boldsymbol{\Phi(t)}$ defines the shape of the input function (see main text)**.** We non-dimensionalize the model by defining:

$\boldsymbol{x=}\frac{\boldsymbol{[X]}}{\boldsymbol{K}_{\boldsymbol{x}}}$**,** $\boldsymbol{r}_{\boldsymbol{1}}\boldsymbol{=}\frac{\boldsymbol{[}\boldsymbol{R}_{\boldsymbol{1}}\boldsymbol{]}\boldsymbol{k}_{\boldsymbol{3}}}{\boldsymbol{K}_{\boldsymbol{x}}\boldsymbol{k}_{\boldsymbol{2}}}$**,** $\boldsymbol{r=}\frac{\boldsymbol{[R]k}_{\boldsymbol{3}}}{\boldsymbol{k}_{\boldsymbol{2}}\boldsymbol{K}_{\boldsymbol{x}}}$**,** $\boldsymbol{\tau=t}\boldsymbol{d}_{\boldsymbol{x}}$**,** $\boldsymbol{\tau}_{\boldsymbol{0}}\boldsymbol{=}\boldsymbol{t}_{\boldsymbol{0}}\boldsymbol{d}_{\boldsymbol{x}}$**,** $\boldsymbol{\gamma}_{\boldsymbol{R}\boldsymbol{1}}\boldsymbol{=}\frac{\boldsymbol{d}_{\boldsymbol{R}\boldsymbol{1}}}{\boldsymbol{d}_{\boldsymbol{x}}}$**,** $\boldsymbol{\gamma}_{\boldsymbol{R}}\boldsymbol{=}\frac{\boldsymbol{d}_{\boldsymbol{R}}}{\boldsymbol{d}_{\boldsymbol{x}}}$**,** $\boldsymbol{\gamma}_{\boldsymbol{o}}\boldsymbol{=}\frac{\boldsymbol{d}_{\boldsymbol{Ro}}}{\boldsymbol{d}_{\boldsymbol{x}}}$**,** $\boldsymbol{\beta''=}\frac{\boldsymbol{k}_{\boldsymbol{3}}}{\boldsymbol{K}_{\boldsymbol{x}}\boldsymbol{d}_{\boldsymbol{x}}}$ **,** and $\boldsymbol{\beta'=}\frac{\boldsymbol{k}_{\boldsymbol{1}}}{\boldsymbol{d}_{\boldsymbol{x}}}$

This leads to the model equations:

$\frac{\boldsymbol{dr}}{\boldsymbol{d\tau}}\boldsymbol{=\beta'}\boldsymbol{r}_{\boldsymbol{1}}\boldsymbol{-}\boldsymbol{(\gamma}_{\boldsymbol{R}}\frac{\boldsymbol{x}^{\boldsymbol{n}}}{\boldsymbol{x}^{\boldsymbol{n}}\boldsymbol{+1}}\boldsymbol{+}\boldsymbol{\gamma}_{\boldsymbol{o}}\boldsymbol{)r}$**, (14)**

$\frac{\boldsymbol{d}\boldsymbol{r}_{\boldsymbol{1}}}{\boldsymbol{d\tau}}\boldsymbol{=}\boldsymbol{\beta}^{\boldsymbol{''}}\boldsymbol{\Phi}\left( \mathsf{\tau} \right)\boldsymbol{-}\boldsymbol{\gamma}_{\boldsymbol{R}\boldsymbol{1}}\boldsymbol{r}_{\boldsymbol{1}}$ **, (15)**

$\frac{\boldsymbol{dx}}{\boldsymbol{d\tau}}\boldsymbol{=\beta''\Phi}\left( \mathsf{\tau} \right)\boldsymbol{-x}$ **, (16)**

As the length of the time delay of the activation arm increases, the ability of $\boldsymbol{R}$ to respond to each pulse is delayed, which limits its capability to exhibit a stepwise increase of the output in response to oscillatory input signals. As a result, increasing the time delay in the manner will reduce the counting quality due to the delay in the production of $\boldsymbol{R}$. However, we demonstrate in S5 Fig that the addition of a sufficiently short time delay in this arm can have little impact on the counting capability and the ability to differentiate between a sustained and a transient input signal.

**S4. Analytical solution of final R**

The dimensionless base model (Equations 1 and 2 in the main text) can be solved analytically for the final level of $\boldsymbol{R}$ under three assumptions:

1. The input pulse ($\boldsymbol{\Phi(}\mathsf{\tau)}$) is a square wave function, with identical pulses that are evenly distributed (Figure 1A).
2. The induced degradation is an all or none response, depending on if $\boldsymbol{X}$ exceeds its threshold concentration.
3. The parameters are such that the counting capacity is maintained (i.e. $\boldsymbol{X}$ always remains below the threshold concentration and is unable to trigger the degradation of $\boldsymbol{R}$). Given assumption (2), $\boldsymbol{X}$ does not contribute to $\boldsymbol{R}$’s degradation.

Under these assumptions, the accumulation of $\boldsymbol{R}$ follows: $\frac{\boldsymbol{dr}}{\boldsymbol{d\tau}}\boldsymbol{=\beta\Phi}\left( \mathsf{\tau} \right)\boldsymbol{-}\boldsymbol{\gamma}_{\boldsymbol{o}}\boldsymbol{r}$ **.** During the ***i^th^*** pulse, $\boldsymbol{R}$ reaches a peak concentration at the end of the pulse, and is degraded until the end of the simulation:

$\boldsymbol{r}_{\boldsymbol{start, i}}\boldsymbol{=}\boldsymbol{r}_{\boldsymbol{end, i-1}}\boldsymbol{e}^{\boldsymbol{-(T-D)}\boldsymbol{\gamma}_{\boldsymbol{o}}}$**, (17)**

$\boldsymbol{r}_{\boldsymbol{end, i}}\boldsymbol{=}\frac{\boldsymbol{\beta}}{\boldsymbol{\gamma}_{\boldsymbol{o}}}\boldsymbol{-(}\frac{\boldsymbol{\beta}}{\boldsymbol{\gamma}_{\boldsymbol{o}}}\boldsymbol{-}\boldsymbol{r}_{\boldsymbol{start, i}}\boldsymbol{)}\boldsymbol{e}^{\boldsymbol{-}\boldsymbol{\gamma}_{\boldsymbol{o}}\boldsymbol{D}}$**, (18)**

where $\boldsymbol{r}_{\boldsymbol{end, i}}$ is the peak concentration of $\boldsymbol{R}$ after the $\boldsymbol{i}^{\boldsymbol{th}}$ pulse and $\boldsymbol{r}_{\boldsymbol{start, i}}$ is the concentration of $\boldsymbol{R}$ immediately before the $\boldsymbol{i}^{\boldsymbol{th}}$ pulse. To start, $\boldsymbol{r}_{\boldsymbol{start, 1}}\boldsymbol{=0}$ while both $\boldsymbol{r}_{\boldsymbol{start, i}}$ and $\boldsymbol{r}_{\boldsymbol{end, i}}$ increase alongside $\boldsymbol{i}$. Using the equations above, we can determine the level of $\boldsymbol{R}$ after $\boldsymbol{k}$ pulses $\boldsymbol{(k>0)}$**,** at time $\mathsf{\tau}_{\boldsymbol{E}}$ ***>*** $\left( \boldsymbol{k-1} \right)\boldsymbol{T+}\mathsf{\tau}_{\boldsymbol{o}}\boldsymbol{+D}$**:**

$\boldsymbol{r}\left( \mathsf{\tau}_{\boldsymbol{E}} \right)\boldsymbol{=}\frac{\boldsymbol{\beta}}{\boldsymbol{\gamma}_{\boldsymbol{o}}}$**(1-**$\boldsymbol{e}^{\mathbf{-}\boldsymbol{\gamma}_{\boldsymbol{o}}\boldsymbol{D}}$**)**$\sum_{\boldsymbol{i=0}}^{\boldsymbol{k-1}} \boldsymbol{e}^{\mathbf{-}\boldsymbol{\gamma}_{\boldsymbol{o}}\boldsymbol{(}\mathsf{\tau}_{\boldsymbol{E}}\boldsymbol{-}\mathsf{\tau}_{\boldsymbol{0}}\boldsymbol{-(iT+D))}}$ **(19)**

**S5. Effects of stochastic dynamics**

The dynamics of a motif in isolation will not necessarily persist when it is placed in the context of a larger network. This is a potential caveat when interpreting any dynamic property of any network motif. With this in mind, we explore the extent by which the counting property can persist in the presence of different perturbations or variations (e.g. cellular noise). The stronger the motif can resist such perturbations, the more likely our counting mechanism can persist in a larger context. The intrinsically random nature of chemical reactions and gene expression result in fluctuations in the molecular number of interacting molecules. This noise is commonly found in natural networks and presents a challenge to reliable cellular function. To test the impact of noise, we implement a stochastic model using equations 5-6 in the main text. We extend the base model into a set of stochastic differential equations (SDEs) to account for both intrinsic and extrinsic noise sources:

$\frac{\boldsymbol{d[R]}}{\boldsymbol{dt}}\boldsymbol{=}\boldsymbol{k}_{\boldsymbol{1}}\boldsymbol{\Phi}\left( \boldsymbol{t} \right)\boldsymbol{-}\boldsymbol{d}_{\boldsymbol{R}} \frac{\left[ \boldsymbol{X} \right]^{\boldsymbol{n}}}{\left[ \boldsymbol{X} \right]^{\boldsymbol{n}}\boldsymbol{+}\boldsymbol{K}_{\boldsymbol{x}}^{\boldsymbol{n}}} \left[ \boldsymbol{R} \right]\boldsymbol{-}\boldsymbol{d}_{\boldsymbol{Ro}}\left[ \boldsymbol{R} \right]\boldsymbol{+}\boldsymbol{\zeta}_{\boldsymbol{1}}\boldsymbol{-}\boldsymbol{\zeta}_{\boldsymbol{2}}\boldsymbol{-}\boldsymbol{\zeta}_{\boldsymbol{3}}\boldsymbol{+\xi}$ **, (20)**

$\frac{\boldsymbol{d[X]}}{\boldsymbol{dt}}\boldsymbol{=}\boldsymbol{k}_{\boldsymbol{2}}\boldsymbol{\Phi}\left( \boldsymbol{t} \right)\boldsymbol{-}\boldsymbol{d}_{\boldsymbol{x}}\left[ \boldsymbol{X} \right]\boldsymbol{+}\boldsymbol{\zeta}_{\boldsymbol{4}}\boldsymbol{-}\boldsymbol{\zeta}_{\boldsymbol{5}}\boldsymbol{+ \xi}$ **, (21)**

Here, we define intrinsic noise ($\boldsymbol{\zeta}_{\boldsymbol{l}}$ **–** associated with a chemical reaction l as defined by Table S1) as noise that arises from the stochastic nature of chemical reactions and extrinsic noise ($\boldsymbol{\xi}$) as noise that originates from cellular machinery outside of the circuit [1]. We solve these SDEs numerically using an Euler method implemented in Dynetica [2]. Our simulations indicate that the presence of noise can reduce the ability to both count and differentiate between transient and sustained signals. As the level of the noise increases, $\boldsymbol{X}$ has an increased likelihood of overcoming its threshold even for a parameter set that satisfies $\boldsymbol{1>\beta(1-}\boldsymbol{e}^{\boldsymbol{-D}}\boldsymbol{)}$ (S8A-B Fig). Similarly, noise can either remove the ability to count or reduce the quality of counting depending on the amplitude of the noise with respect to the molecular number (S8 Fig). With a sufficiently large molecular number, however, counting can be maintained in the presence of both intrinsic and extrinsic noise (S8C Fig).

**Table S1. Noise sources and corresponding reactions**

| **Noise Source** | **Reaction** | **Formulation** |
| --- | --- | --- |
| $\boldsymbol{\zeta}_{\boldsymbol{1}}$ | **Production of** $\boldsymbol{R}$ | $\sqrt{\boldsymbol{k}_{\boldsymbol{1}}\boldsymbol{\Phi(t)}}\boldsymbol{Г}_{\boldsymbol{1}}$ |
| $\boldsymbol{\zeta}_{\boldsymbol{2}}$ | **Induced degradation of** $\boldsymbol{R}$ | $\sqrt{\boldsymbol{d}_{\boldsymbol{R}} \frac{\left[ \boldsymbol{X} \right]^{\boldsymbol{n}}}{\left[ \boldsymbol{X} \right]^{\boldsymbol{n}}\boldsymbol{+}\boldsymbol{K}_{\boldsymbol{x}}^{\boldsymbol{n}}} \left[ \boldsymbol{R} \right]}\boldsymbol{Г}_{\boldsymbol{2}}$ |
| $\boldsymbol{\zeta}_{\boldsymbol{3}}$ | **Endogenous degradation of** $\boldsymbol{R}$ | $\sqrt{\boldsymbol{d}_{\boldsymbol{Ro}}\left[ \boldsymbol{R} \right]}\boldsymbol{Г}_{\boldsymbol{3}}$ |
| $\boldsymbol{\zeta}_{\boldsymbol{4}}$ | **Production of** $\boldsymbol{X}$ | $\sqrt{\boldsymbol{k}_{\boldsymbol{2}}\boldsymbol{\Phi(t)}}\boldsymbol{Г}_{\boldsymbol{4}}$ |
| $\boldsymbol{\zeta}_{\boldsymbol{5}}$ | **Endogenous degradation of** $\boldsymbol{X}$ | $\sqrt{\boldsymbol{d}_{\boldsymbol{x}}\boldsymbol{[X]}}\boldsymbol{Г}_{\boldsymbol{5}}$ |

**(**$\boldsymbol{Г}_{\boldsymbol{i}}$**’s are independent Gaussian noise)**

**S6. Alternative model: repression of R synthesis by X**

Here, we implement an alternative model where the intermediate represses the production of $\boldsymbol{R}$ rather than induce its degradation. We use the following system of ODEs to define the network:

$\frac{\boldsymbol{d[R]}}{\boldsymbol{dt}}\boldsymbol{=}\boldsymbol{k}_{\boldsymbol{1}}\frac{{\boldsymbol{K}_{\boldsymbol{x}}}^{\boldsymbol{n}}}{\left[ \boldsymbol{X} \right]^{\boldsymbol{n}}\boldsymbol{+}\boldsymbol{K}_{\boldsymbol{x}}^{\boldsymbol{n}}}\boldsymbol{\Phi(t)} \boldsymbol{-}\boldsymbol{d}_{\boldsymbol{Ro}}\boldsymbol{[R]}$ **, (22)**

$\frac{\boldsymbol{d[X]}}{\boldsymbol{dt}}\boldsymbol{=}\boldsymbol{k}_{\boldsymbol{2}}\boldsymbol{\Phi(t)} \boldsymbol{-}\boldsymbol{d}_{\boldsymbol{x}}\boldsymbol{[X]}$ **, (23)**

where $\boldsymbol{k}_{\boldsymbol{1}}$ and $\boldsymbol{k}_{\boldsymbol{2}}$ are the rate constants for the production of $\boldsymbol{R}$ and $\boldsymbol{X}$ respectively, and $\boldsymbol{d}_{\boldsymbol{x}}$ and $\boldsymbol{d}_{\boldsymbol{Ro}}$ are the rate constants for the endogenous degradation of $\boldsymbol{X}$ and $\boldsymbol{R}$ respectively. The term $\frac{{\boldsymbol{K}_{\boldsymbol{x}}}^{\boldsymbol{n}}}{\left[ \boldsymbol{X} \right]^{\boldsymbol{n}}\boldsymbol{+}\boldsymbol{K}_{\boldsymbol{x}}^{\boldsymbol{n}}}$ represents the threshold response through Hill kinetics for the repression of the production of $\boldsymbol{R}$ by $\boldsymbol{X}$ (S9 Fig). $\boldsymbol{\Phi(t)}$ defines the shape of the input function (see main text)**.**

We non-dimensionalize the model by defining:

$\boldsymbol{x=}\frac{\boldsymbol{[X]}}{\boldsymbol{K}_{\boldsymbol{x}}}$, $\boldsymbol{r=}\frac{\boldsymbol{[R]k}_{\boldsymbol{2}}}{\boldsymbol{k}_{\boldsymbol{1}}\boldsymbol{K}_{\boldsymbol{x}}}$, $\boldsymbol{\tau=t}\boldsymbol{d}_{\boldsymbol{x}}$, $\boldsymbol{\tau}_{\boldsymbol{0}}\boldsymbol{=}\boldsymbol{t}_{\boldsymbol{0}}\boldsymbol{d}_{\boldsymbol{x}}$, $\boldsymbol{\gamma}_{\boldsymbol{o}}\boldsymbol{=}\frac{\boldsymbol{d}_{\boldsymbol{Ro}}}{\boldsymbol{d}_{\boldsymbol{x}}}$, and $\boldsymbol{\beta=}\frac{\boldsymbol{k}_{\boldsymbol{2}}}{\boldsymbol{K}_{\boldsymbol{x}}\boldsymbol{d}_{\boldsymbol{x}}}$

This leads to the following equations:

$\frac{\boldsymbol{dr}}{\boldsymbol{d\tau}}\boldsymbol{=\beta}\frac{\boldsymbol{1}}{\boldsymbol{x}^{\boldsymbol{n}}\boldsymbol{+1}}\boldsymbol{\Phi(}\mathsf{\tau)-}\boldsymbol{\gamma}_{\boldsymbol{o}}\boldsymbol{r}$**, (24)**

$\frac{\boldsymbol{dx}}{\boldsymbol{d\tau}}\boldsymbol{=\beta\Phi(}\mathsf{\tau)-}\boldsymbol{x}$**, (25)**

With this model, as $\boldsymbol{x}$ increases alongside an increasing $\boldsymbol{D}$, the production of $\boldsymbol{R}$ is suppressed through Hill kinetics. Here, $\boldsymbol{X}$ does not need to reach some threshold level before affecting the suppression of $\boldsymbol{R}$. Rather, higher concentrations of $\boldsymbol{X}$ increase the strength of the repression on the production of $\boldsymbol{R}$. As a result, increasing $\boldsymbol{\beta}$ increases the ability to differentiate between transient and sustained signals. And like the model in the main text, increasing $\boldsymbol{\gamma}_{\boldsymbol{o}}$ reduces the counting quality by degrading the response of $\boldsymbol{R}$ to the input signal. S9 Fig shows that, with appropriate parameters, the system maintains the ability to differentiate between a transient and sustained signal as well as count in the case of an oscillatory signal.

**Supplementary Figures**

**S1 Fig.** **Incoherent Feedforward Loop and pulse counting.**

**(A)** **Time courses for pulses defined by a sine function.** This panel uses $\beta$=1.2,$\gamma_{R}$=10, $\gamma_{O}$=0, and $T$ = 4 (Left: $D$ = 1 Right: $D$ = 3). The top row contains the time courses of the input pulses for two different pulse durations, either a pulsing input or a simulated sustained input (with a sine waveform). The second row shows the time courses for $\boldsymbol{X}$. The bottom row shows the time courses for $\boldsymbol{R}$. With identical parameters, an IFFL motif can generate two distinct outputs depending on the length of the duration of the input pulses. When $\boldsymbol{X}$ is below the threshold of induction, the circuit maintains the ability to produce a stepwise increase of $\mathbf{R}$. However, when $\boldsymbol{X}$ overcomes the threshold, the circuit loses this ability. For a single parameter set, both outputs are desired to quantify the system as being capable of counting.

**(B)** **Calibration curve for ideal counting.** This panel uses $\beta$=1.2,$\gamma_{R}$=10, $\gamma_{O}$=0, and $T$ = 4 ($D$ = 1). The sample calibration curve is for a pulse duration within the optimal duration range, therefore it is able to demonstrate ideal counting. With an increasing number of input pulses from 1-4 in the top panel, $\boldsymbol{R}$ exhibits a stepwise increase. The linearity is demonstrated in the bottom panel by **R^2^ > 0.99**.

**S2 Fig. Failure in counting.**

Here, A and B both use $\beta$=1.2,$\gamma_{R}$=10, $\gamma_{O}$=0, and $T$ = 4 ($D$ = 3), a parameter set which demonstrates the inability to count.

**(A) Time course for failed counting.** With an increasing number of pulses, $\boldsymbol{X}$ overcomes the activation threshold and the ability to produce a stepwise increase of $\boldsymbol{R}$ is lost.

**(B)** **Calibration curve for failed counting.** The sample calibration curve is for a pulse duration outside the optimal duration range, therefore it is able to demonstrate the case when counting fails. With an increasing number of input pulses from 1-10 $\boldsymbol{r}$ is suppressed and the non-linearity is demonstrated by **R^2^ < 0.99**.

**S3 Fig. Counting with the alternative model**

**(A)** **Alternative incoherent feedforward loop motif (Model in S1).** In this model, a pulsing input simultaneously stimulates the production of $\boldsymbol{X}$ and $\boldsymbol{R}$. Here, the threshold response is implemented with the additional production of $\boldsymbol{X}$ through a positive feedback loop.

**(B)** **Calibration curves for ideal or failed counting.**

**The top panel uses** $\boldsymbol{\beta}$**=1.2,**$\boldsymbol{\gamma}_{\boldsymbol{R}}$**=0.01,** $\boldsymbol{\gamma}_{\boldsymbol{O}}$**=0, *α*=100, and** $\boldsymbol{T}$ **= 10 (**$\boldsymbol{D}$ **= 1).** The sample calibration curve is for a pulse duration within the optimal duration range, therefore it demonstrates counting.

**The bottom panel uses** $\boldsymbol{\beta}$**=1.2,**$\boldsymbol{\gamma}_{\boldsymbol{R}}$**=0.01,** $\boldsymbol{\gamma}_{\boldsymbol{O}}$**=0, *α*=100, and** $\boldsymbol{T}$ **= 10** **(**$\boldsymbol{D}$ **= 9).** The sample calibration curve is for a pulse duration outside the optimal duration range, therefore it demonstrates the case when counting fails.

**S4 Fig. An IFFL can maintain robust counting of oscillating signals in the presence of additional time delay in the inhibition arm.**

**(A)** **Signal processing by an IFFL (Model in S2).** In this model, a pulsing input **(**$\boldsymbol{S}$**)** simultaneously stimulates the production of an intermediate **(**$\boldsymbol{X}_{\boldsymbol{1}}$***)*** and a reporter **(**$\boldsymbol{R}$**)**. The first intermediate **(**$\boldsymbol{X}_{\boldsymbol{1}}$**)** then activates the production of the second intermediate **(**$\boldsymbol{X}$**)** that induces the degradation of $\boldsymbol{R}$ through a threshold response. The fundamental constraints for counting shown are based on the full model (S1 Text).

**(B)** **Time courses demonstrate counting mechanism.** Using $\beta'$=1, $\beta''$=1.2,$\gamma_{R}$=10, $\delta$=1, $\gamma_{O}$=0, and $T$ = 4 (Left: $D$ = 1 Right: $D$ = 3). The top row contains time courses of the input pulses for two different pulse durations, either a pulsing input or a simulated sustained input. The second row shows time courses for $\boldsymbol{X}_{\boldsymbol{1}}$*.* The third row shows time courses for $\boldsymbol{X}$. The bottom row shows time courses for $\boldsymbol{R}$. Despite the addition of time delay (of ~10% of the pulse duration) through an additional component, the IFFL motif maintains the ability to distinguish input signals with longer or shorter pulses.

**S5 Fig. An IFFL can maintain robust counting of oscillating signals in the presence of additional time delay in the activation arm.**

**(A)** **Signal processing by an IFFL (Model in S3).** In this model, a pulsing input ($\boldsymbol{S}$) simultaneously stimulates the production of an intermediate $(\boldsymbol{X}\boldsymbol{)}$ and an intermediate reporter ${\boldsymbol{(}\boldsymbol{R}}_{\boldsymbol{1}\boldsymbol{)}}$. The intermediate reporter ${\boldsymbol{(}\boldsymbol{R}}_{\boldsymbol{1}}\boldsymbol{)}$ then activates the production of the reporter $(\boldsymbol{R}\boldsymbol{)}$, which is degraded by the intermediate $\boldsymbol{(}\boldsymbol{X}\boldsymbol{)}$ through a threshold response. The fundamental constraints for counting shown are based on the full model (S1 Text).

**(B)** **Time courses demonstrate counting mechanism.** Using $\beta'$=5, $\beta''$=1.2,$\gamma_{R}$=10, $\gamma_{R1}$=5, $\gamma_{O}$=0, and $T$ = 4 (Left: $D$ = 1 Right: $D$ = 3). The top row contains time courses of the input pulses for two different pulse durations, either a pulsing input or a simulated sustained input. The second row shows time courses for $\boldsymbol{X}$. The third row shows time courses for $\boldsymbol{R}_{\boldsymbol{1}}$. The bottom row shows time courses for $\boldsymbol{R}$. Despite the addition of time delay (of ~10% of the pulse duration) through an additional component, the IFFL motif maintains the ability to distinguish input signals with longer or shorter pulses.

**S6 Fig. Distinguishing between a sustained input and an oscillatory input that are on average at the same level.**

**(A)** **Time courses demonstrate counting mechanism.** Using $\beta$=1.2,$\gamma_{R}$=10, $\gamma_{O}$=0, and $T$ = 4 (Left: $D$ = 1 Right: $D$ = 3). The top row contains time courses of the input pulses for two different pulse durations, either a pulsing input or a simulated sustained input. The second row shows time courses for $\boldsymbol{X}$. The bottom row shows time courses for $\boldsymbol{R}$.

**(B)** **Time courses demonstrate differentiation between a sustained and oscillatory input.** Using $\beta$= 0.337*,*$\gamma_{R}$=10, $\gamma_{O}$=0. Here, the amplitude of the sustained input is equivalent to the mean of the oscillating input in panel A ($D$ = 1).

**S7 Fig. Ultrasensitivity (as indicated by a high Hill coefficient) in induced degradation is critical for robust counting.**

**Time courses of the reporter.** Using $\beta$=1.2,$\gamma_{R}$=10, $\gamma_{O}$=0, $T$ = 4, $D$ = 1. Here, we show time courses for $\boldsymbol{R}$ with varying values for the Hill coefficient (n = 5, 50, 110, or 1000) to demonstrate that the specific value for n is irrelevant. The hill coefficient must simply be high enough to induce a threshold response.

**S8 Fig. Stochastic simulations of IFFL**

**(A)** **Time courses demonstrate the effect of noise in the case of a small molecular number (Model S5).** Using $k_{1}=k_{2}=5$, $d_{x}=1$, $d_{R}=10$, n = 100, $d_{Ro}=0$, $K_{x}=4$, and $T$ = 4 ($D$ = 1) in equations 20-21. The top row shows time courses of the input pulses in the case of an oscillating input. The second row shows time courses for $\boldsymbol{R}$ in the absence of noise. The third row shows time courses for $\boldsymbol{X}$ in the presence of noise ($\boldsymbol{\xi}$ = 10). The fourth row shows time courses for $\boldsymbol{R}$ in the presence of noise. The bottom row shows the calibration curve for the system in the presence of noise. With an increasing number of input pulses from 1-10, $\boldsymbol{R}$ is unable to exhibit a stepwise increase due to the impact of noise on the low molecular number of the components; this poor linearity is demonstrated by **R^2^ < 0.99**.

**(B)** **Time courses demonstrate the effect of noise in the case of an increasing molecular number (Model S5).** Using $k_{1}=k_{2}=50$, $d_{x}=1$, $d_{R}=10$, n = 100, $d_{Ro}=0$, $K_{x}=40$, and $T$ = 4 ($D$ = 1) in equations 20-21. The top row contains time courses of the input pulses in the case of an oscillating input. The second row shows time courses for $\boldsymbol{R}$ in the absence of noise. The third row shows time courses for $\boldsymbol{X}$ in the presence of noise ($\boldsymbol{\xi}$= 10). The fourth row shows time courses for $\boldsymbol{R}$ in the presence of noise. The bottom row shows the calibration curve for the system in the presence of noise. With an increasing number of input pulses from 1-10, $\boldsymbol{R}$ is unable to exhibit a stepwise increase due to the impact of noise on the low molecular number of the components; the poor linearity is demonstrated by **R^2^ < 0.99**.

**(C)** **Time courses demonstrate the effect of noise in the case of a sufficiently high molecular number (Model S5).** Using $k_{1}=k_{2}=500$, $d_{x}=1$, $d_{R}=10$, n = 100, $d_{Ro}=0$, $K_{x}=400$, and $T$ = 4 ($D$ = 1) in equations 20-21. The top row contains time courses of the input pulses in the case of an oscillating input. The second row shows time courses for $\boldsymbol{R}$ in the absence of noise. The third row shows time courses for $\boldsymbol{X}$ in the presence of noise ($\boldsymbol{\xi}$= 10). The fourth row shows time courses for $\boldsymbol{R}$ in the presence of noise. The bottom row shows the calibration curve for the system in the presence of noise. With an increasing number of input pulses from 1-10, $\boldsymbol{R}$ is able to exhibit a stepwise increase despite the presence of noise due to the sufficiently high molecular number. Here, the linearity is demonstrated by **R^2^ ≥ 0.99**.

**S9 Fig. The implementation of the threshold response in an IFFL through repression.**

**(A)** **Signal processing by an IFFL (Model S6).** In this model, a pulsing input $(\boldsymbol{S}\boldsymbol{)}$ simultaneously stimulates the production of an intermediate $\boldsymbol{(}\boldsymbol{X}\boldsymbol{)}$ and a reporter $\boldsymbol{(}\boldsymbol{R}\boldsymbol{)}$. The intermediate represses the production of $\boldsymbol{R}$ through a threshold response. The fundamental constraints for counting shown are based on the model described in S1 Text.

**(B)** **Time courses demonstrate counting mechanism.** Using $\beta$=12, $\gamma_{O}$=0, and $T$ = 4 (Left: $D$ = 1 Right: $D$ = 3). The top row contains time courses of the input pulses for two different pulse durations, either a pulsing input or a simulated sustained input. The second row shows time courses for $\boldsymbol{X}$. The bottom row shows time courses for $\boldsymbol{R}$. Here, an IFFL motif can generate two distinct outputs depending on the length of the duration of the input pulses.

**Reference**

1. Gillespie DT (2000) The chemical Langevin equation. Journal of Chemical Physics 113: 297.
2. Eidum D, Asthana K, Unni S, Deng M, You LC. Construction, visualization, and analysis of biological network models in Dynetica[J]. Quant. Biol., 2014, 2(4): 142-150.
