# Supplementary figures and images for: Processing Oscillatory Signals by Incoherent Feedforward Loops
Source: PLoS Comput Biol. 2016 Sep 13;12(9):e1005101. doi: 10.1371/journal.pcbi.1005101 (PMC5021367; doi:10.1371/journal.pcbi.1005101)

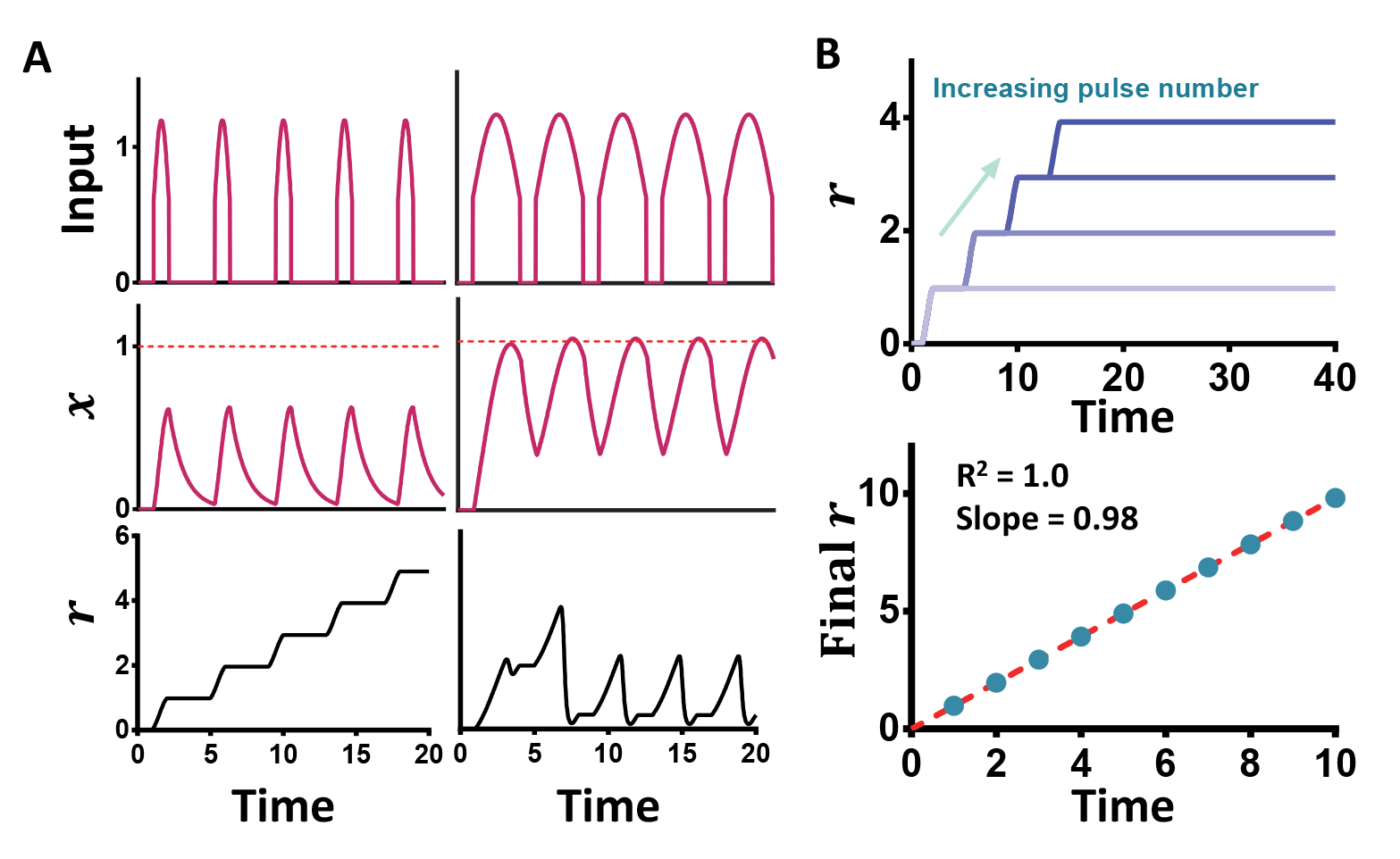

Supplement: S1 Fig — (A) Time courses for pulses defined by a sine function. This panel uses β = 1.2, γR = 10, γO = 0, and T = 4 (Left: D = 1 Right: D = 3). The top row contains the time courses of the input pulses for two different pulse durations, either a pulsing input or a simulated sustained input (with a sine waveform). The second row shows the time courses for X. The bottom row shows the time courses for R. With identical parameters, an IFFL motif can generate two distinct outputs depending on the length of the duration of the input pulses. When X is below the threshold of induction, the circuit maintains the ability to produce a stepwise increase of R. However, when X overcomes the threshold, the circuit loses this ability. For a single parameter set, both outputs are desired to quantify the system as being capable of counting. (B) Calibration curve for ideal counting. This panel uses β = 1.2, γR = 10, γO = 0, and T = 4 (D = 1). The sample calibration curve is for a pulse duration within the optimal duration range, therefore it is able to demonstrate ideal counting. With an increasing number of input pulses from 1–4 in the top panel, R exhibits a stepwise increase. The linearity is demonstrated in the bottom panel by R2 > 0.99. (TIF) [file pcbi.1005101.s002.tif]

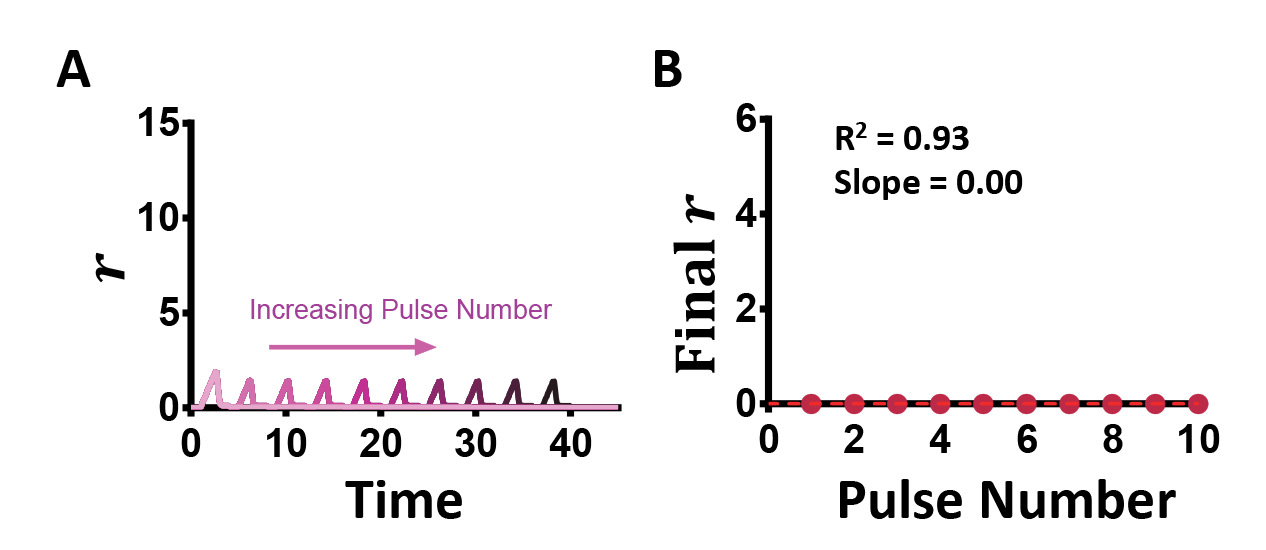

Supplement: S2 Fig — Here, A and B both use β = 1.2, γR = 10, γO = 0, and T = 4 (D = 3), a parameter set which demonstrates the inability to count. (A) Time course for failed counting. With an increasing number of pulses, X overcomes the activation threshold and the ability to produce a stepwise increase of R is lost. (B) Calibration curve for failed counting. The sample calibration curve is for a pulse duration outside the optimal duration range, therefore it is able to demonstrate the case when counting fails. With an increasing number of input pulses from 1–10 r is suppressed and the non-linearity is demonstrated by R2 < 0.99. (TIF) [file pcbi.1005101.s003.tif]

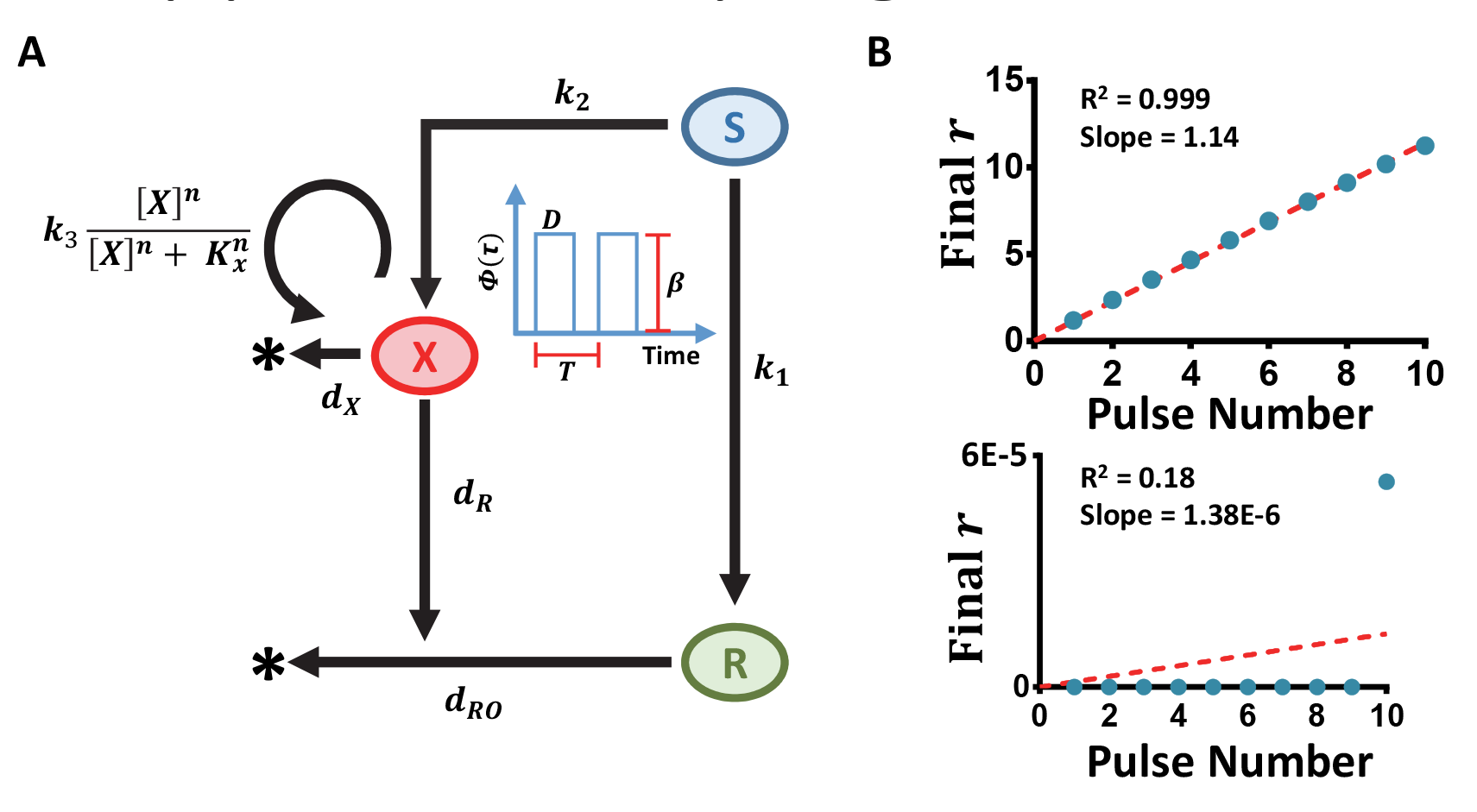

Supplement: S3 Fig — (A) Alternative incoherent feedforward loop motif (Model in S1). In this model, a pulsing input simultaneously stimulates the production of X and R. Here, the threshold response is implemented with the additional production of X through a positive feedback loop. (B) Calibration curves for ideal or failed counting. The top panel uses β = 1.2, γR = 0.01, γO = 0, α = 100, and T = 10 (D = 1). The sample calibration curve is for a pulse duration within the optimal duration range, therefore it demonstrates counting. The bottom panel uses β = 1.2, γR = 0.01, γO = 0, α = 100, and T = 10 (D = 9). The sample calibration curve is for a pulse duration outside the optimal duration range, therefore it demonstrates the case when counting fails. (TIF) [file pcbi.1005101.s004.tif]

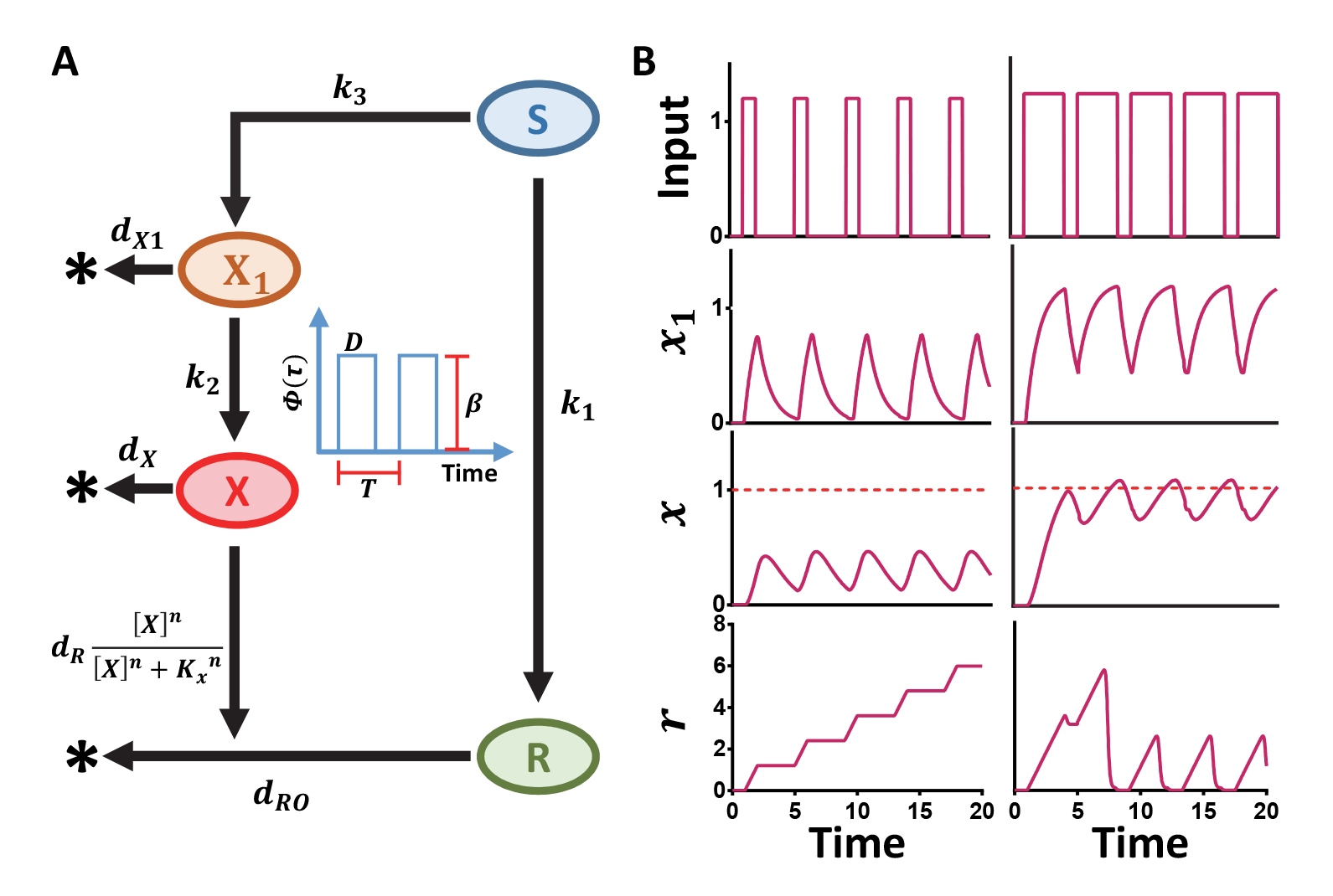

Supplement: S4 Fig — (A) Signal processing by an IFFL (Model in S2). In this model, a pulsing input (S) simultaneously stimulates the production of an intermediate (X1) and a reporter (R). The first intermediate (X1) then activates the production of the second intermediate (X) that induces the degradation of R through a threshold response. The fundamental constraints for counting shown are based on the full model (S1 Text). (B) Time courses demonstrate counting mechanism. Using β′ = 1, β″ = 1.2, γR = 10, δ = 1, γO = 0, and T = 4 (Left: D = 1 Right: D = 3). The top row contains time courses of the input pulses for two different pulse durations, either a pulsing input or a simulated sustained input. The second row shows time courses for X1. The third row shows time courses for X. The bottom row shows time courses for R. Despite the addition of time delay (of ~10% of the pulse duration) through an additional component, the IFFL motif maintains the ability to distinguish input signals with longer or shorter pulses. (TIF) [file pcbi.1005101.s005.tif]

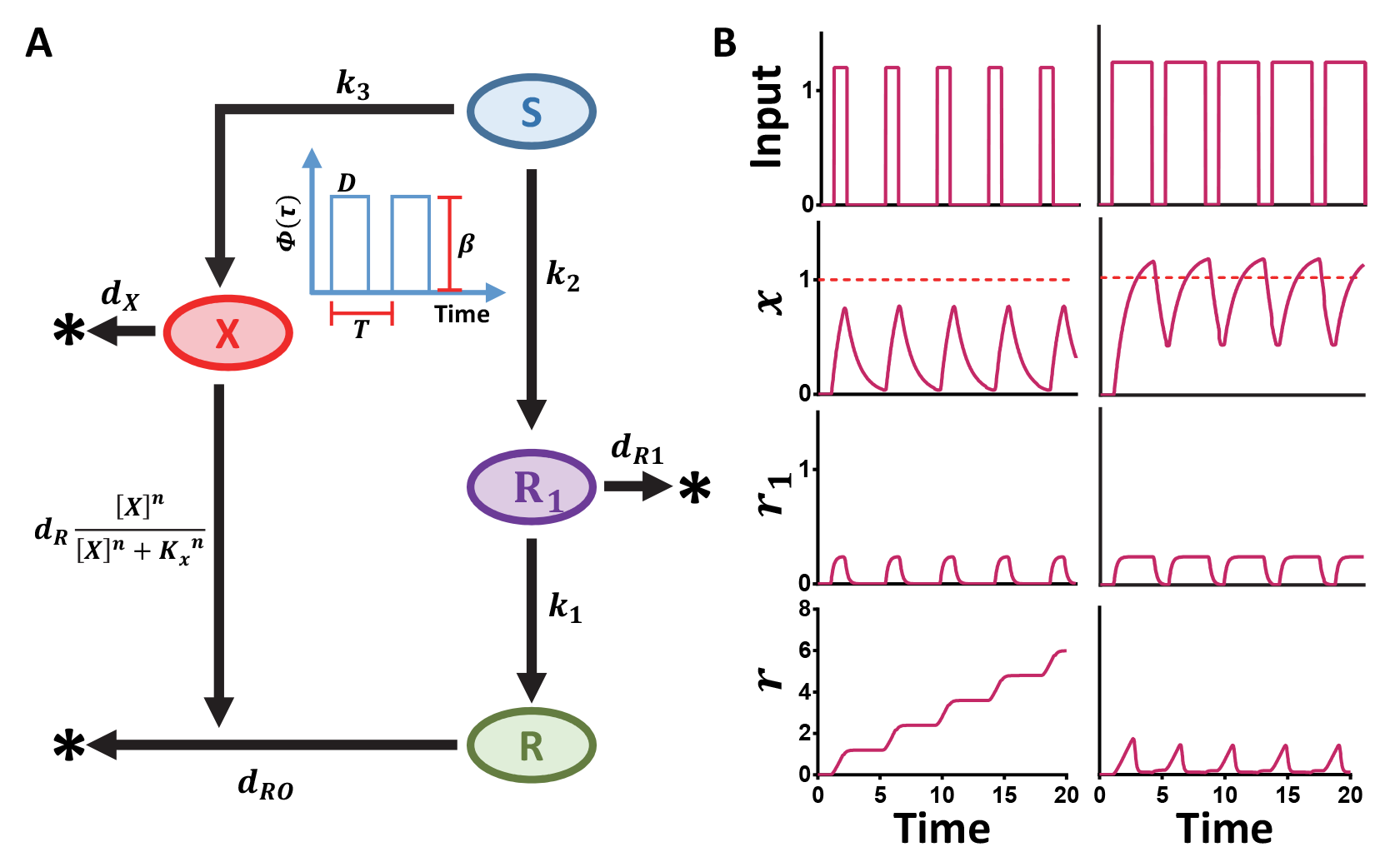

Supplement: S5 Fig — (A) Signal processing by an IFFL (Model in S3). In this model, a pulsing input (S) simultaneously stimulates the production of an intermediate (X) and an intermediate reporter (R1). The intermediate reporter (R1) then activates the production of the reporter (R), which is degraded by the intermediate (X) through a threshold response. The fundamental constraints for counting shown are based on the full model (S1 Text). (B) Time courses demonstrate counting mechanism. Using β′ = 5, β″ = 1.2, γR = 10, γR1 = 5, γO = 0, and T = 4 (Left: D = 1 Right: D = 3). The top row contains time courses of the input pulses for two different pulse durations, either a pulsing input or a simulated sustained input. The second row shows time courses for X. The third row shows time courses for R1. The bottom row shows time courses for R. Despite the addition of time delay (of ~10% of the pulse duration) through an additional component, the IFFL motif maintains the ability to distinguish input signals with longer or shorter pulses. (TIF) [file pcbi.1005101.s006.tif]

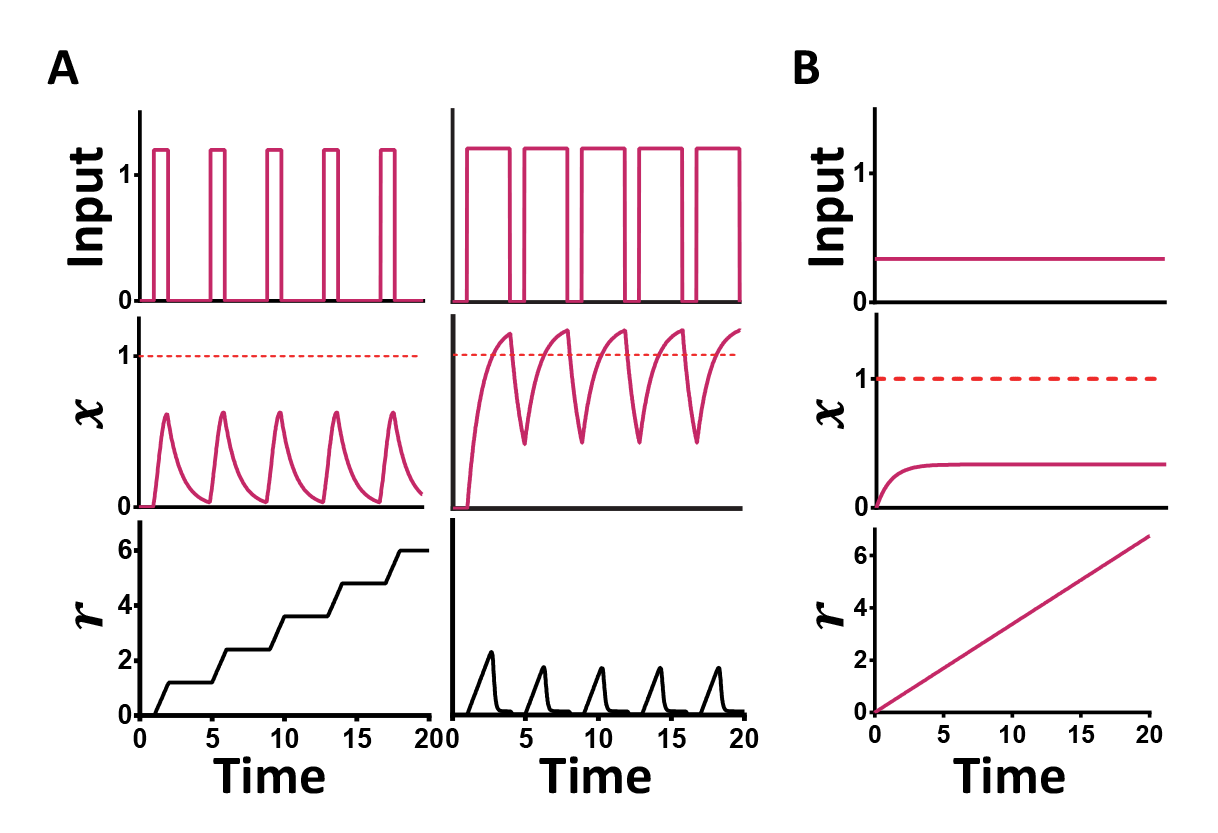

Supplement: S6 Fig — (A) Time courses demonstrate counting mechanism. Using β = 1.2, γR = 10, γO = 0, and T = 4 (Left: D = 1 Right: D = 3). The top row contains time courses of the input pulses for two different pulse durations, either a pulsing input or a simulated sustained input. The second row shows time courses for X. The bottom row shows time courses for R. (B) Time courses demonstrate differentiation between a sustained and oscillatory input. Using β = 0.337, γR = 10, γO = 0. Here, the amplitude of the sustained input is equivalent to the mean of the oscillating input in panel A (D = 1). (TIF) [file pcbi.1005101.s007.tif]

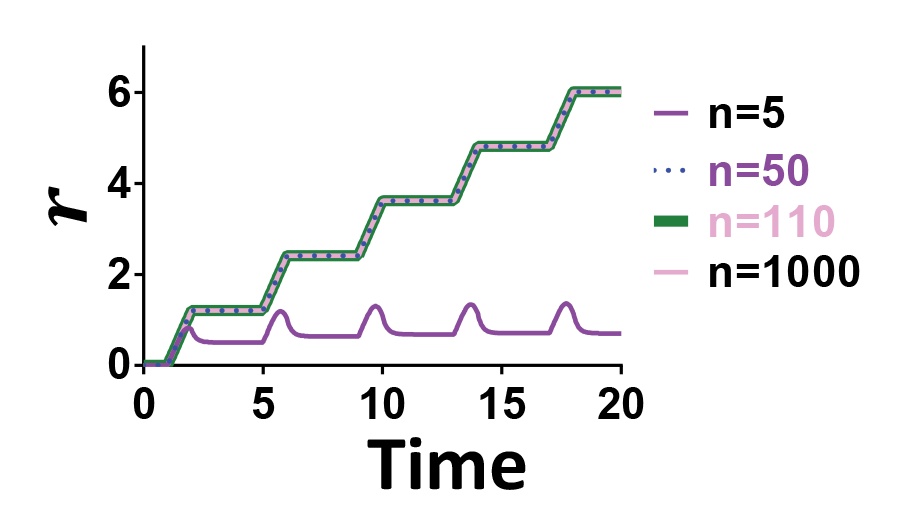

Supplement: S7 Fig — Time courses of the reporter. Using β = 1.2, γR = 10, γO = 0, T = 4, D = 1. Here, we show time courses for R with varying values for the Hill coefficient (n = 5, 50, 110, or 1000) to demonstrate that the specific value for n is irrelevant. The hill coefficient must simply be high enough to induce a threshold response. (TIF) [file pcbi.1005101.s008.tif]

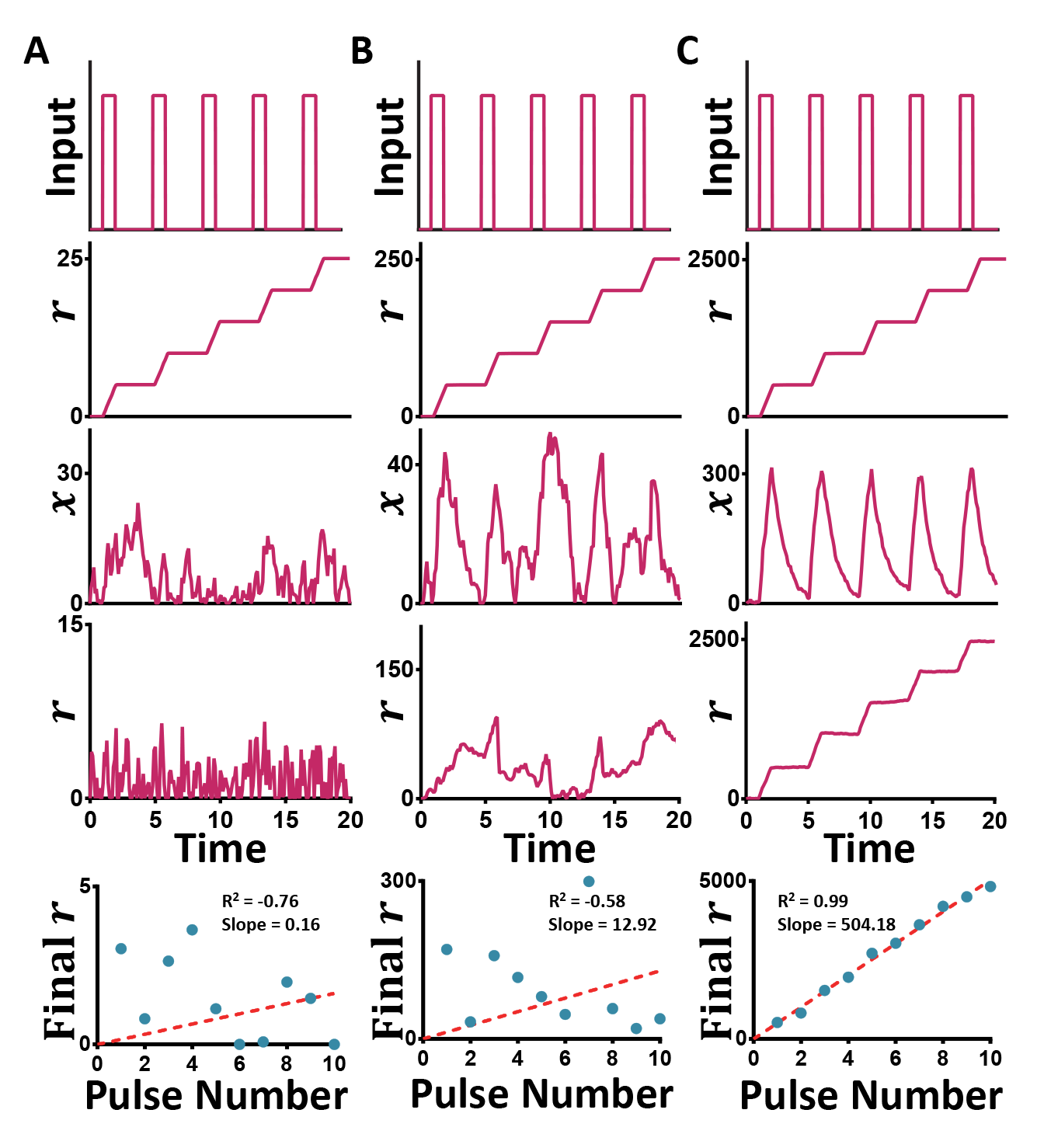

Supplement: S8 Fig — (A) Time courses demonstrate the effect of noise in the case of a small molecular number (Model S5). Using k1 = k2 = 5, dx = 1, dR = 10, n = 100, dRo = 0, Kx = 4, and T = 4 (D = 1) in equations 20–21. The top row shows time courses of the input pulses in the case of an oscillating input. The second row shows time courses for R in the absence of noise. The third row shows time courses for X in the presence of noise (ξ = 10). The fourth row shows time courses for R in the presence of noise. The bottom row shows the calibration curve for the system in the presence of noise. With an increasing number of input pulses from 1–10, R is unable to exhibit a stepwise increase due to the impact of noise on the low molecular number of the components; this poor linearity is demonstrated by R2 < 0.99. (B) Time courses demonstrate the effect of noise in the case of an increasing molecular number (Model S5). Using k1 = k2 = 50, dx = 1, dR = 10, n = 100, dRo = 0, Kx = 40, and T = 4 (D = 1) in equations 20–21. The top row contains time courses of the input pulses in the case of an oscillating input. The second row shows time courses for R in the absence of noise. The third row shows time courses for X in the presence of noise (ξ = 10). The fourth row shows time courses for R in the presence of noise. The bottom row shows the calibration curve for the system in the presence of noise. With an increasing number of input pulses from 1–10, R is unable to exhibit a stepwise increase due to the impact of noise on the low molecular number of the components; the poor linearity is demonstrated by R2 < 0.99. (C) Time courses demonstrate the effect of noise in the case of a sufficiently high molecular number (Model S5). Using k1 = k2 = 500, dx = 1, dR = 10, n = 100, dRo = 0, Kx = 400, and T = 4 (D = 1) in equations 20–21. The top row contains time courses of the input pulses in the case of an oscillating input. The second row shows time courses for R in the absence of noise. The third row shows t [file pcbi.1005101.s009.tif]

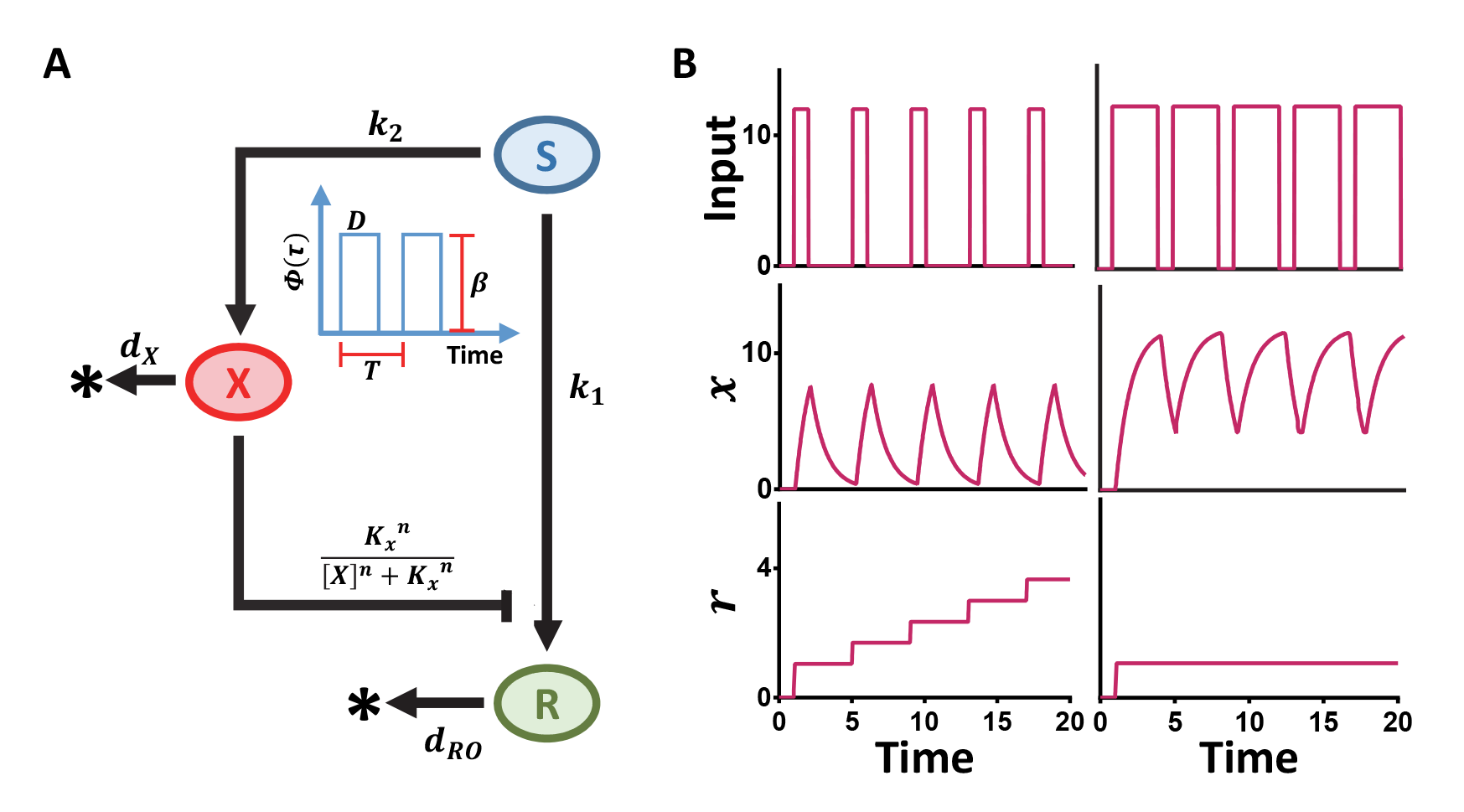

Supplement: S9 Fig — (A) Signal processing by an IFFL (Model S6). In this model, a pulsing input (S) simultaneously stimulates the production of an intermediate (X) and a reporter (R). The intermediate represses the production of R through a threshold response. The fundamental constraints for counting shown are based on the model described in S1 Text. (B) Time courses demonstrate counting mechanism. Using β = 12, γO = 0, and T = 4 (Left: D = 1 Right: D = 3). The top row contains time courses of the input pulses for two different pulse durations, either a pulsing input or a simulated sustained input. The second row shows time courses for X. The bottom row shows time courses for R. Here, an IFFL motif can generate two distinct outputs depending on the length of the duration of the input pulses. (TIF) [file pcbi.1005101.s010.tif]
